# Supplementary material for: Enhanced cell deconvolution of peripheral blood using DNA methylation for high-resolution immune profiling
Source: Nat Commun. 2022 Feb 9;13:761. doi: 10.1038/s41467-021-27864-7 (PMC8828780; doi:10.1038/s41467-021-27864-7)
Supplement: Supplementary file 1 — Supplementary material [file 41467_2021_27864_MOESM1_ESM.pdf]

Supplementary material

Enhanced cell deconvolution of peripheral blood using DNA methylation for high-resolution immune profiling

Lucas A Salas[1], Ze Zhang[1], Devin C Koestler[2], Rondi A Butler[3], Helen M Hansen[4], Annette M Molinaro[4], John K Wiencke[4,5], Karl T Kelsey[3]*, Brock C Christensen[1,6]*

*Correspondence: Brock.C.Christensen@dartmouth.edu

Affiliations

[1]Department of Epidemiology, Geisel School of Medicine, Dartmouth College, Lebanon, NH, USA.

[2]Department of Biostatistics & Data Science, University of Kansas Medical Center, Kansas City, KS, USA.

[3]Departments of Epidemiology and Pathology and Laboratory Medicine, Brown University, Providence, RI, USA.

[4]Department of Neurological Surgery, University of California San Francisco, San Francisco, CA, USA.

[5]Institute for Human Genetics, University of California San Francisco, San Francisco, CA, USA.

[6]Departments of Molecular and Systems Biology, and Community and Family Medicine, Geisel School of Medicine, Dartmouth College, Lebanon, NH, USA.

Table of Contents

Supplementary Table 1 Cell-type definitions according to flow cytometry markers 3

Supplementary Table 2 Demographic characteristics of the donors for the cell isolated samples 4

Supplementary Fig. 1. Cell purity estimated by flow sorting verification (a), and estimated DNA methylation purity (b) 5

Supplementary Fig. 2. Known SNPs tracing to genetic ancestry markers distribution across the cell-types in the libraries 6

Supplementary Fig. 3. Principal component regression analysis of phenotype and technical variables for the samples included in the library... 7

Supplementary Table 3. Dirichlet distributions of artificial mixtures used for training, testing, and independent validation for IDOL... 8

Supplementary Table 4. Comparison of different sizes of optimal libraries using IDOL for EPIC technology, and the legacy IDOL in the 450k common probes 9

Supplementary Table 5. Enrichment of genomic context areas in the libraries vs. the background probes used for selection after exclusion of cross reactive, polymorphic, CpH and sex chromosomes 10

Supplementary Fig. 4. Comparison of the EPIC IDOL-ext, 450k IDOL-Ext and minfi pickCompProbes automatic selection estimations per cell-type. Automatic selection is severely biased for T-cells subtypes, Bcell naïve and eosinophils 11

Supplementary Fig. 5. Comparison between cell proportion estimations from the EPIC IDOL-ext (panel a) and cell counts estimations (panel b) in whole blood DNA samples with FCM data. 12

Supplementary Fig. 6. Validation of additional components of the EPIC IDOL-ext (a) and 450k IDOL-ext (b) libraries using flow cytometry 13

Supplementary Fig. 7. Exploratory analysis applying the libraries to umbilical cord blood datasets. 14

Supplementary Fig. 8. eForge 2.0 enrichment for differentially methylated regions ENCODE H3K4me1 (primed enhancers) 15

Supplementary Table 6. Baseline characteristics of the samples included in the application datasets from GEO and ArrayExpress 16

Supplementary Fig. 9. Predicted immune cell proportions in whole blood samples between multiple sclerosis cases (n=13) and normal controls (n=14) (450k) 17

Supplementary Fig. 10. Predicted immune cell proportions in peripheral blood leukocyte samples between rheumatoid arthritis cases (n=354) and normal controls (n=335) (450k) 18

Supplementary Fig. 11. Predicted immune cell proportions in peripheral blood samples from early breast cancer patients before and after receiving radiation therapy only (a n=74) and radiation therapy plus chemotherapy (b, n=70) (EPIC) 19

Supplementary Fig. 12. Changes in estimated immune cell proportions between subjects with COVID-19 infection with and without remission compared to healthy subjects (EPIC) 20

Supplementary Fig. 13. Differences of predicted immune cell proportions in whole blood samples between pairs of twins in monozygotic twins (n=852) and dizygotic twins (n=612) (450k) 21

Supplementary Fig. 14. Trajectories of several cell subpopulation ratios across different ages (in years) using publicly available datasets (n=2504) 22

Supplementary Fig. 15. Changes in predicted immune cell proportions with aging using samples from ages zero to greater than 90 years (n=2504) (450k and EPIC) 23

Supplementary Fig. 16. Longitudinal changes of predicted immune cell proportions between 3 months to 5 years after birth in human blood leukocytes of 10 healthy girls 24

Supplementary Fig. 17. Bland Altman plots comparing the estimation using constrained projection/quadratic programming (CP/QP) versus the true values using the EPIC IDOL-Ext and the 450k IDOL-Ext libraries 25

Supplementary Fig. 18. Bland Altman plots comparing three different statistical deconvolution methods (CIBERSORT- CBS, constrained projection/quadratic programming-CP/QP, robust partial correlations-RPC) estimates versus the true values using the EPIC IDOL-Ext library 26

Supplementary Table 1 Cell-type definitions according to flow cytometry markers

Group	Subgroup	Cell-type	Abbreviation	Selection	Markers	Source
Myeloid	Mononuclear cells	Monocytes (classical)	Mono	Negative	CD14(+)	PBMC
	Granulocytes	Basophils	Bas	Negative	IgE(+) CD123(+)	PMN rich fraction basophil labelling system
		Eosinophils	Eos	Negative	CD15(+) CD16(-)	PMN rich fraction (HetaSep)
		Neutrophils	Neu	Negative	NA	PMN rich fraction (HetaSep)
Lymphoid	T-cells	T regulatory cells	Treg	Negative	CD4(+) CD25(+) CD127dim/(-)	PBMC
		T helper CD4+ naive cells	CD4nv	Negative	CD4(+) CD45RA(+) CD45RO(-)	PBMC
		T helper CD4+ memory cells	CD4mem	Negative	CD4(+) CD45RA(-) CD45RO(+)	PBMC
		T cytotoxic CD8+ naive cells	CD8nv	Negative	CD8(+) CD45RA(+) CCR7(+) CD45RO(-) CD56(-) CD57(-)	PBMC
		T cytotoxic CD8+ memory cells (effector memory)	CD8mem	Positive	CD8(+) CD45RO(+) CD62(-)	PBMC
	B-cells	B naïve cells	Bnv	Negative	CD19(+) CD27(-)	PBMC
		B memory cells	Bmem	Positive	CD19(+) CD27(+)	PBMC
	Natural killers	Natural killers	NK	Negative	CD56(+)	PBMC

Supplementary Table 2 Demographic characteristics of the donors for the cell isolated samples

Dataset (Platform)	N	Mean Age (SD)	N Male (%)	N European* (%)	N African* (%)	N East Asian* (%)
Discovery Datasets (EPIC)						
Basophil	6	45.67 (13.95)	5 (83.33)	5 (83.33)	1 (16.67)	0 (0)
B naïve	4	34.5 (5.26)	2 (50)	3 (75)	1 (25)	0 (0)
B memory	6	32 (12.02)	3 (50)	5 (83.33)	1 (16.67)	0 (0)
CD4 naïve	5	24.4 (2.3)	5 (100)	5 (100)	0 (0)	0 (0)
CD4 memory	4	41 (14.49)	3 (75)	4 (100)	0 (0)	0 (0)
CD8 naïve	5	29.4 (9.86)	4 (80)	5 (100)	0 (0)	0 (0)
CD8 memory	4	25.5 (3.79)	2 (50)	4 (100)	0 (0)	0 (0)
Eosinophil	4	24.75 (4.5)	3 (75)	0 (0)	2 (50)	2 (50)
Monocyte	5	33 (9.77)	4 (80)	3 (60)	1 (20)	1 (20)
Neutrophil	6	26.83 (7.25)	5 (83.33)	5 (83.3)	1 (16.7)	0 (0)
NK	4	33.75 (13.45)	4 (100)	4 (100)	0 (0)	0 (0)
T regulatory	3	34.33 (15.50)	1 (33.33)	1 (33.3)	2 (66.7)	0 (0)

All the data is deposited in GEO (GSE167998). *Modified names categorization based on the sesame genetic ancestry algorithm instead of self-reporting (the algorithm was trained using TCGA race self-report categories). When compared to self-reported ethnicity, European ancestry includes subjects from multiple Indo-European ancestry ethnic groups (e.g., Americans and Europeans, South Asia, Hispanics/Latinxs, and African-Americans admixed populations). See Supplementary Fig. 2 for additional details. This algorithm does not include other categories or subgroups.

Supplementary Fig. 1. Cell purity estimated by flow sorting verification (a), and estimated DNA methylation purity (b)

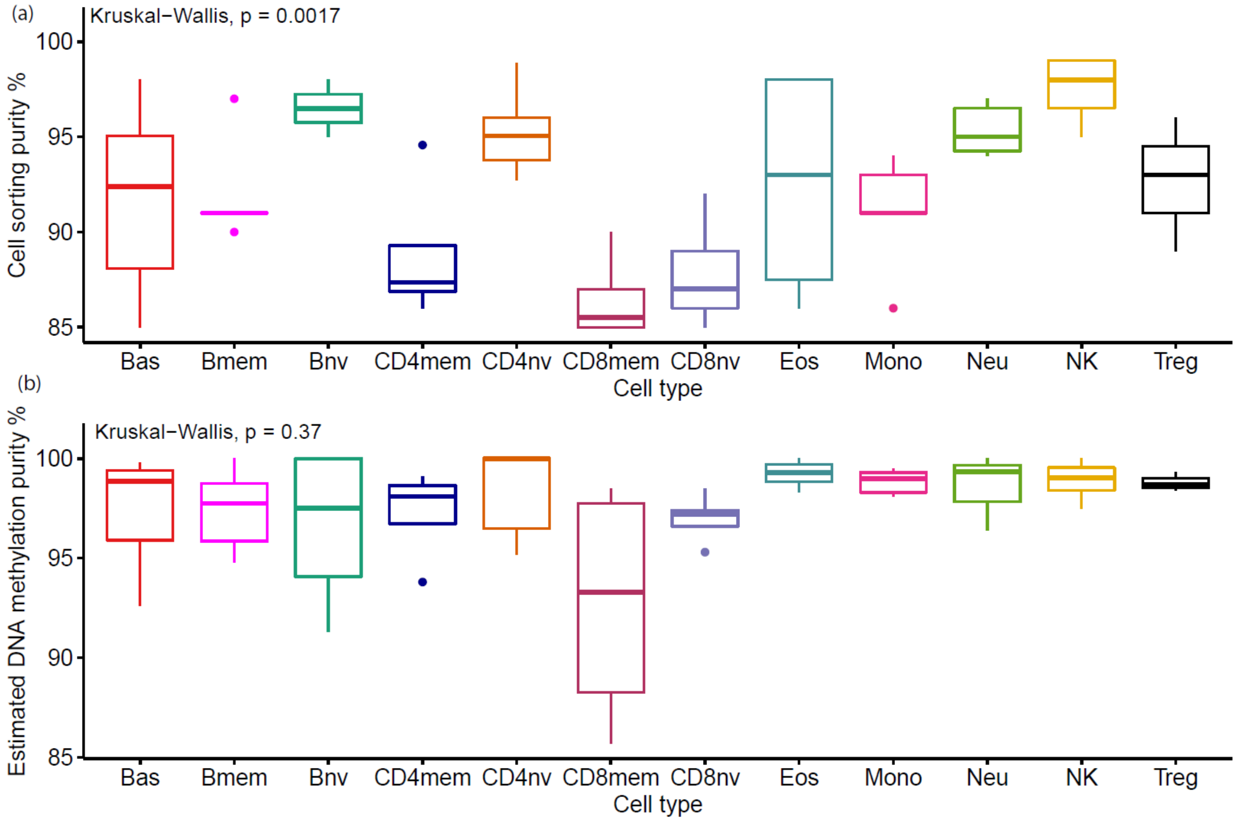

The boxplots include the following information: (1) The box shows the interquartile range (IQR), (2) the whiskers show the inner fences (1.5 × IQR out of the box), (3) the bolded line shows the median of the data. The color of the box corresponds to the cell-type. A two-sided Kruskall-Wallis test was used to compare the median of the distributions. Data are available at GSE167998. See Supplementary Table 1 for the abbreviations. Source data are provided as a Source Data file.

Supplementary Fig. 2. Known SNPs tracing to genetic ancestry markers distribution across the cell-types in the libraries

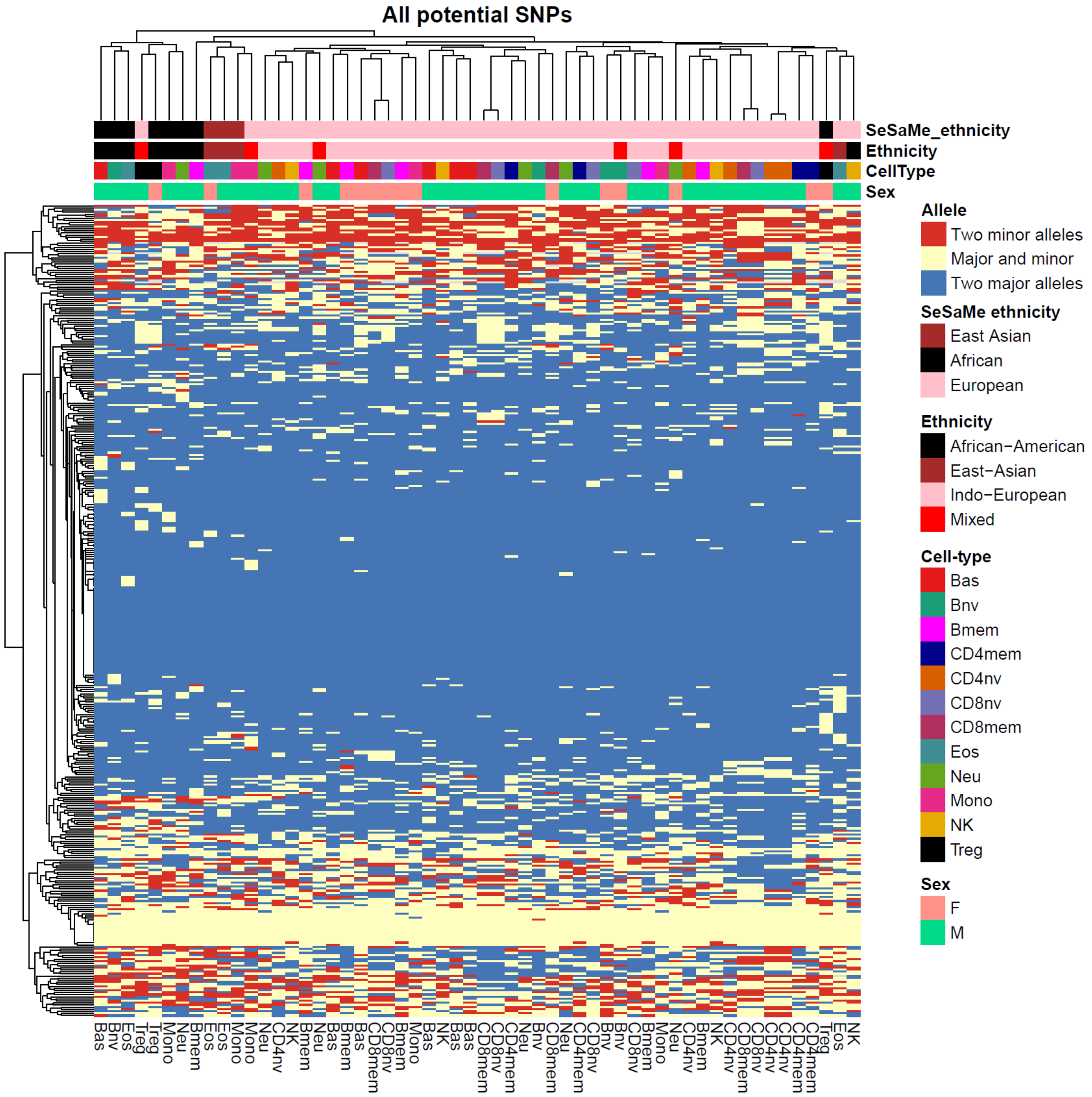

Major and minor alleles were determined using the in-band allele (Zhou et al.). Although named "ethnicity," SeSaMe uses SNP ancestry markers to group the subjects using a random forest approach; the names reported here were modified from the program's output (the algorithm is trained based on TCGA race self-report, see Zhou et al. 2017 for details). Ethnicity is grouped into major ancestry groups based on the ethnicity self-report of the donors. Here the category of African-American describes subjects who self-reported their race as Black and were born in the USA and potentially with different unknown degrees of ancestry admixture. The Mixed category includes subjects reporting multiple ethnicities and those reported as Hispanic/Latinos and potentially with an unknown ancestry admixture. Data are available at GSE167998. See Supplementary Table 1 for the abbreviations. Source data are provided as a Source Data file.

Supplementary Fig. 3. Principal component regression analysis of phenotype and technical variables for the samples included in the library

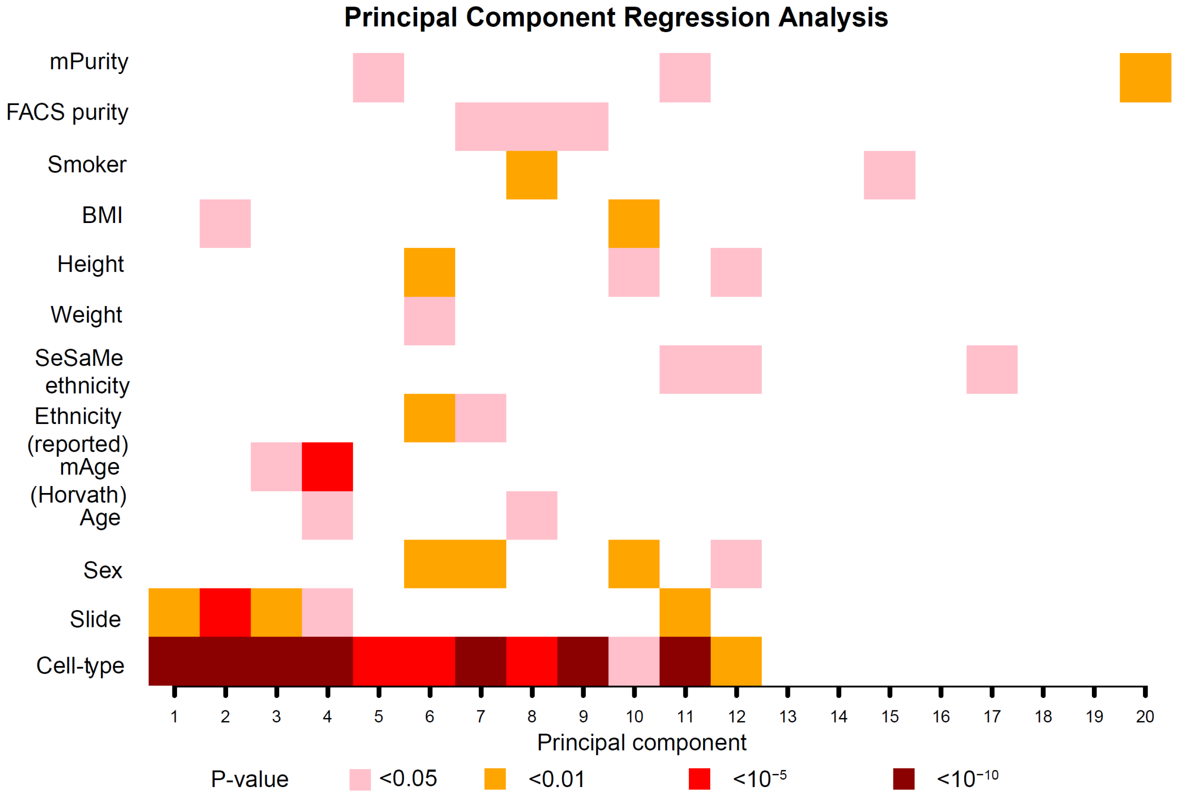

mPurity: methylation purity derived from the automatic deconvolution by Jaffe et al. FACS purity: reported flow sorting estimates of purity. BMI: body mass index (continuous). SeSaMe "ethnicity": refers to single nucleotide polymorphisms ancestry markers, and the random forest approach by Zhou et al. Ethnicity (reported) is based on self-reported ethnicity of the donor. mAge (Horvath): methylation age using Horvath's epigenetic clock. The p-values are derived from the F-test for the linear model using the principal component as the outcome and the phenotype interrogated as the explanatory variable, one variable per time. The p-value was not adjusted for multiple comparisons. Data can be derived from GSE167998. See Supplementary Table 1 for the abbreviations. Source data are provided as a Source Data file.

Supplementary Table 3. Dirichlet distributions of artificial mixtures used for training, testing, and independent validation for IDOL.

	Bas	Bmem	Bnv	CD4mem	CD4nv	CD8mem	CD8nv	Eos	Mono	Neu	NK	Treg
Training												
MIX_1	0	8.4	14.1	13	5.7	7.7	13.2	3.9	8.2	8.8	17	0
MIX_2	0	13.3	7.7	7.8	16.8	5.4	5.9	20	6.5	5.8	10.8	0
MIX_3	9.4	4.4	6.4	20.8	10.8	8.2	8	6.5	9.5	8.2	7.8	0
MIX_4	10.4	8.7	23.8	8.9	8.8	5.5	5.5	6.5	5.5	7.5	8.9	0
MIX_5	0	11.2	14.4	0	8	8.2	5.6	10.8	18.1	12	5.7	6
MIX_6	0	7.5	11	0	9.9	11.6	8.7	10.6	5.2	15.4	9	11.1
Testing												
MIX_7	0	5.1	14.7	0	3.3	8.4	5.6	22.7	10.2	7.2	6.8	16
MIX_8	0	16.3	9.7	0	18.4	6.5	9.3	12.9	2.2	7.5	4.7	12.5
MIX_9	0	7.3	8.7	5.4	14	7.7	7.3	16.2	3.2	4.6	10.4	15.2
MIX_10	0	16.5	11.3	8.2	10.5	9.2	6.2	5.8	6	3.7	12.5	10.1
MIX_11	8	7.4	4.3	16.3	8.9	9.4	8.9	9.7	9.7	7.8	6	3.6
MIX_12	13.2	3.6	12.4	11.4	14.8	3.9	7.3	8.6	5.3	6.7	2.8	10
Validation												
MIX_13	8.7	11.1	3.7	21.3	10.2	11.8	2.8	5.1	3.9	3.6	5.1	12.7
MIX_14	0	8	10.3	2.7	9.1	9.8	14.1	16.1	9	11	6	4
MIX_15	7	5.2	5.2	6	7.6	8.3	8.2	5.1	8.1	8.3	22.2	8.8
MIX_16	2.3	7.9	6.5	8.5	9.6	9.5	10	11.3	13.3	9.2	4.5	7.5
MIX_17	13.1	13.3	6.5	10.1	4.5	11	6	9	6.9	3	6.4	10.3
MIX_18	5.6	5.4	7.6	6.2	4.2	5.1	3.8	8	10.6	15.7	16.9	11
MIX_19	9.4	12.5	7.1	8.1	5.1	4.9	6.8	10.4	5	15.7	9.7	5.2
MIX_20	7.4	7.8	11.2	14.1	8.4	6.5	1.9	10.3	6.4	13.7	8.2	4.1
MIX_21	8.5	7.2	18.8	4.7	7.9	9.8	14.8	7	5.8	3.9	7.4	4.2
MIX_22	0	5.2	13.4	12.9	12.3	14.5	7.5	6.9	7.3	8.3	7	4.7
MIX_23	11.5	8.6	6.7	8.7	8.1	9.1	6.2	8.6	3.7	8.6	8.4	11.7
MIX_24	5.1	7.5	6.7	7.2	11.9	9.4	10	7.2	11	9.9	5.1	9.1

Training, testing, and independent validation datasets are deposited in GEO (GSE167998, GSE182379). See Supplementary Table 1 for the abbreviations.

Supplementary Table 4. Comparison of different sizes of optimal libraries using IDOL for EPIC technology, and the legacy IDOL in the 450k common probes

	EPIC IDOL-Ext		450k IDOL-Ext	
	RMSE	R^2	RMSE	R^0
CpG_250	0.631	1	0.612	1
CpG_300	0.526	1	0.505	1
CpG_350	0.499	1	0.608	1
CpG_400	0.404	1	0.373	1
CpG_450	0.342	1	0.394	1
CpG_500	0.338	1	0.255	1
CpG_550	0.342	1	0.344	1
CpG_600	0.307	1	0.235	1
CpG_650	0.293	1	0.249	1
CpG_700	0.446	1	0.233	1
CpG_750	0.281	1	0.232	1
CpG_800	0.323	1	0.299	1
CpG_850	0.274	1	0.296	1
CpG_900	0.277	1	0.295	1
CpG_950	0.250	1	0.212	1
CpG_1000	0.259	1	0.202	1
CpG_1050	0.238	1	0.205	1
CpG_1100	0.254	1	0.198	1
CpG_1200	**0.226**	**1**	0.219	1
CpG_1300	0.255	1	0.498	1
CpG_1400	0.260	1	0.186	1
CpG_1500	0.231	1	**0.175**	**1**
CpG_1600	0.252	1	0.205	1
CpG_1700	0.242	1	0.304	1
CpG_1800	0.235	1	0.211	1
CpG_1900	0.257	1	0.389	1
CpG_2000	0.45	1	0.339	1
CpG_2500	0.466	1	0.368	1
CpG_3000	1.088	0.999	0.705	1

RMSE (root mean square error), R^2 (coefficient of determination). The bolded cells are the selected optimal IDOL libraries.

Supplementary Table 5. Enrichment of genomic context areas in the libraries vs. the background probes used for selection after exclusion of cross reactive, polymorphic, CpH and sex chromosomes

	EPIC IDOL-Ext n=1200 OR (95% CI)	450k IDOL-Ext n=1500 OR (95% CI)	EPIC IDOL-6 n=450 OR (95% CI)	pickCompProbes n=1200 OR (95% CI)
Genomic context				
CpG Island	0.17 (0.12, 0.22)*	0.34 (0.28, 0.41)*	0.10 (0.05, 0.18)*	0.24 (0.18, 0.31)*
Shores	0.83 (0.71, 0.97)*	1.55 (1.38, 1.74)*	0.75 (0.56, 0.98)*	0.64 (0.53, 0.76)*
Shelves	1.17 (0.94, 1.44)	2.26 (1.94, 2.61)*	1.10 (0.76, 1.55)	1.06 (0.84, 1.31)
Open Sea	2.19 (1.93, 2.49)*	0.91 (0.82, 1.00)	2.45 (1.97, 3.07)*	2.34 (2.05, 2.68)*
Functional context				
Promoter	0.52 (0.46, 0.60)*	1.04 (0.94, 1.16)	0.57 (0.45, 0.71)*	0.55 (0.48, 0.63)*
Exon	0.92 (0.73, 1.14)	1.24 (1.03, 1.47)*	0.73 (0.46, 1.09)	1.12 (0.91, 1.38)
Intron	1.88 (1.67, 2.10)*	1.30 (1.17, 1.45)*	1.76 (1.45, 2.13)*	1.83 (1.63, 2.06)*
Intergenic	0.95 (0.83, 1.09)	0.59 (0.51, 0.67)*	1.01 (0.81, 1.25)	0.85 (0.74, 0.97)
Enhancers	3.87 (3.22, 4.62)*	1.57 (1.23, 1.98)*	5.63 (4.30, 7.28)*	5.92 (5.04, 6.91)*
DHS	1.61 (1.42, 1.83)*	1.30 (1.17, 1.45)*	2.00 (1.62, 2.48)*	1.94 (1.71, 2.21)*
Open chromatin	0.79 (0.65, 0.94)*	0.82 (0.69, 0.96)*	0.66 (0.47, 0.91)*	0.65 (0.53, 0.79)*
TFBS	0.89 (0.75, 1.05)	0.82 (0.70, 0.95)*	0.82 (0.61, 1.08)	0.76 (0.64, 0.91)*

*P-value<0.05

Acronyms: OR odds ratio, CI confidence interval, DHS DNase Hypersensitive site, TFBS transcription factor binding site, Ref reference forcomparison in the column, Enhancers Phantom5 enhancers. Genomic context, Enhancers, DHS, and open chromatin information were extracted from the Illumina EPIC annotation file *"IlluminaHumanMethylationEPICanno.ilm10b5.hg38"*. Functional context information was extracted from the UCSC reference genome file *"UCSC_hg19_refGene.bed"*. Source data are provided as a Source Data file.

Supplementary Fig. 4. Comparison of the EPIC IDOL-ext, 450k IDOL-Ext and minfi pickCompProbes automatic selection estimations per cell-type. Automatic selection is severely biased for T-cells subtypes, Bcell naïve and eosinophils

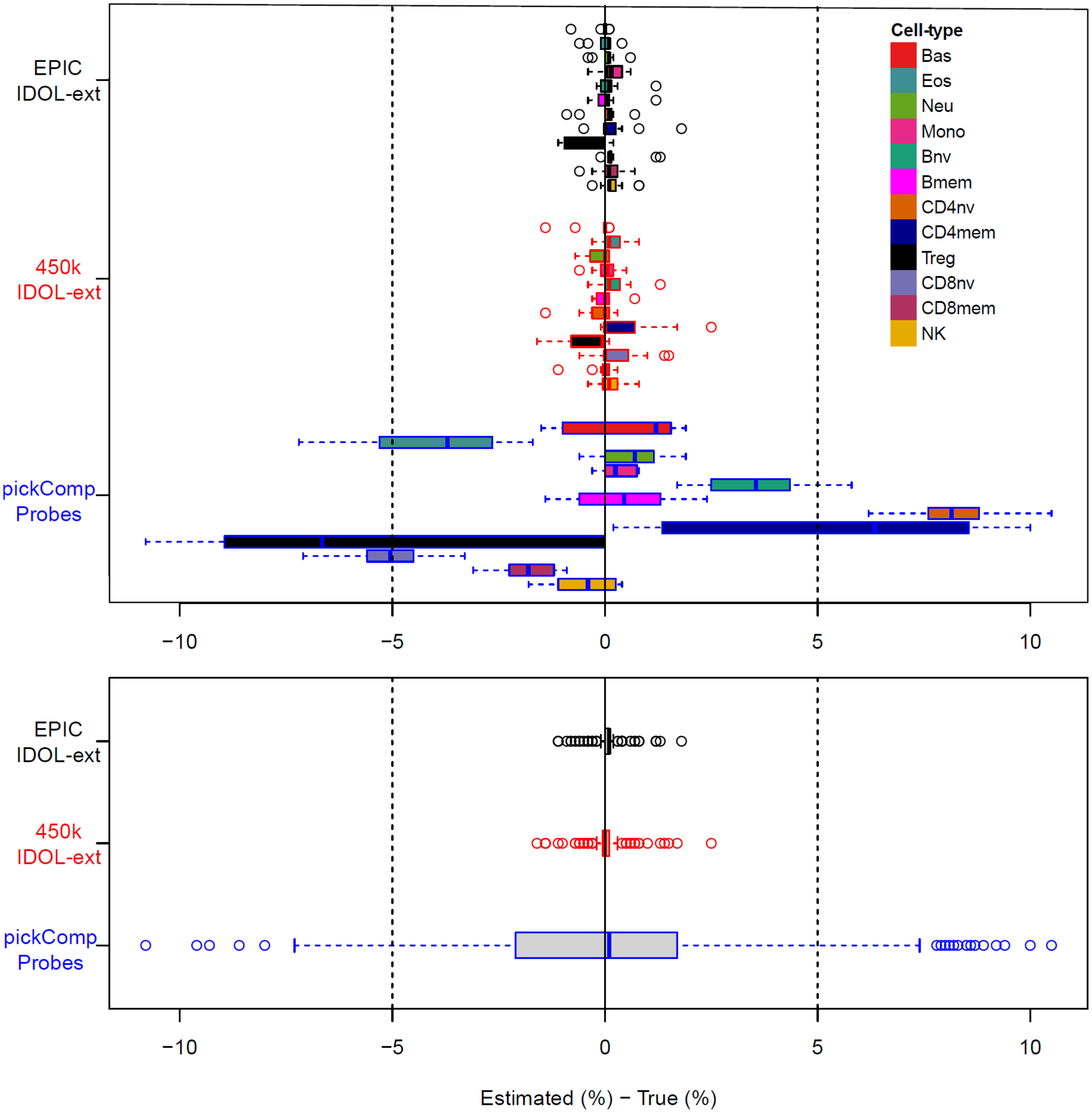

The color of the box corresponds to the library selection method. For the top panel, colors inside the box correspond to the cell type in the same order as the legend of the right side. The boxplots include the following information: (1) The box shows the interquartile range (IQR), (2) the whiskers show the inner fences (1.5 × IQR out of the box), (3) the bolded line shows the median of the data. Data can be derived from GSE167998. See Supplementary Table 1 for the abbreviations. Source data are provided as a Source Data file.

Supplementary Fig. 5. Comparison between cell proportion estimations from the EPIC IDOL-ext (panel a) and cell counts estimations (panel b) in whole blood DNA samples with FCM data.

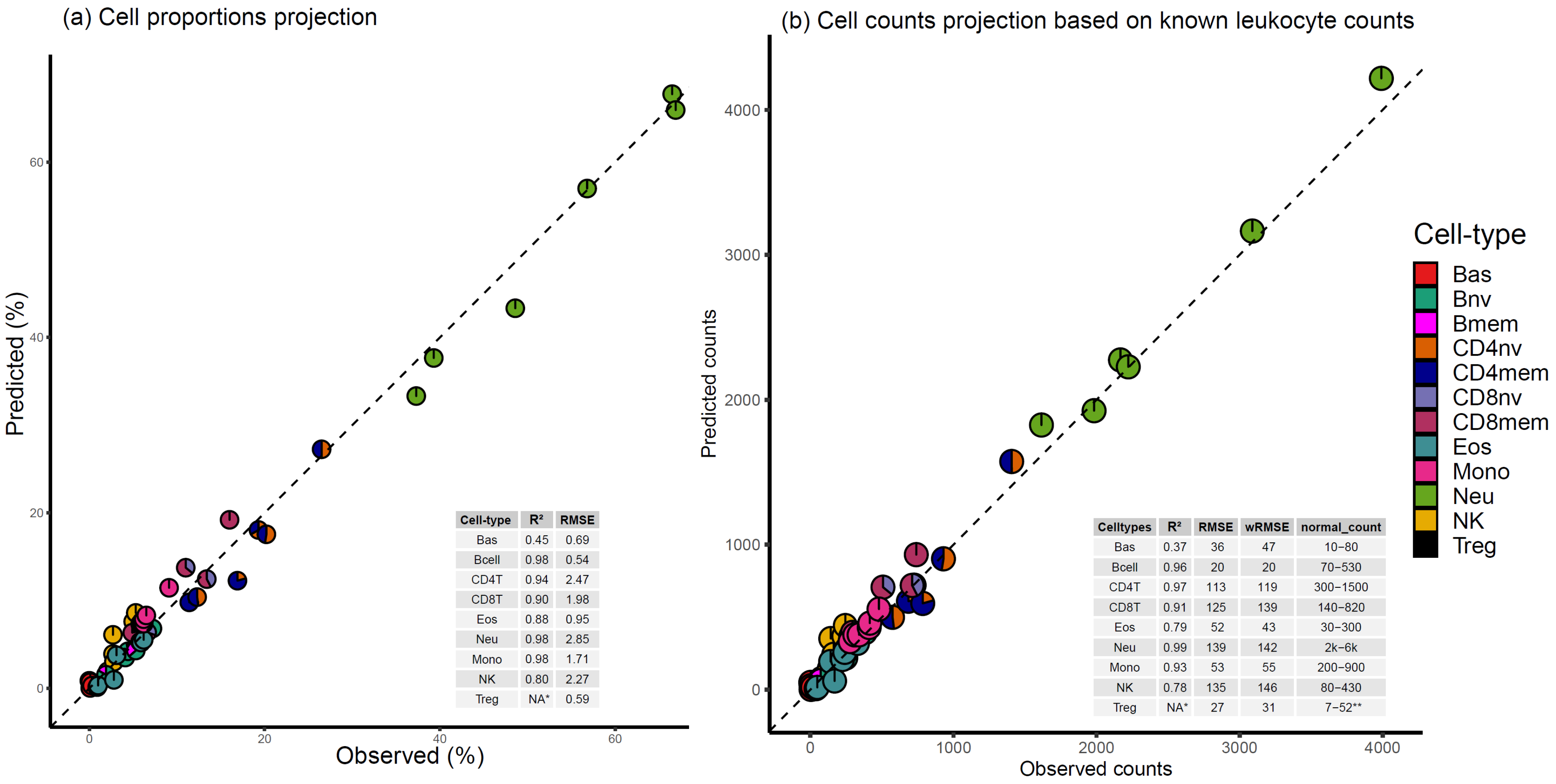

The area of each pieplot corresponds to the estimated proportion of the cell-types within each group. CD4Tcorresponds to the sum of CD4+ T cells naïve, memory, Treg were measured independently. CD8Tcorresponds to the sum of CD8+ T cells naïve and memory. Bcell to the sum of the naïve and memory. Panel B: a measure of weighted root mean square error was added according to the true (FCM) proportions of the measured cells. Normal counts are based on information from STEM-cells. *Estimated Tregs were essentially zeroes, so the R^2 was unstable. **The normal range of Tregs is not established, so the range presented could not reflect the actual counts in peripheral blood. Data are available at GSE110530. See Supplementary Table 1 for the abbreviations. Source data are provided as a Source Data file.

Supplementary Fig. 6. Validation of additional components of the EPIC IDOL-ext (a) and 450k IDOL-ext (b) libraries using flow cytometry

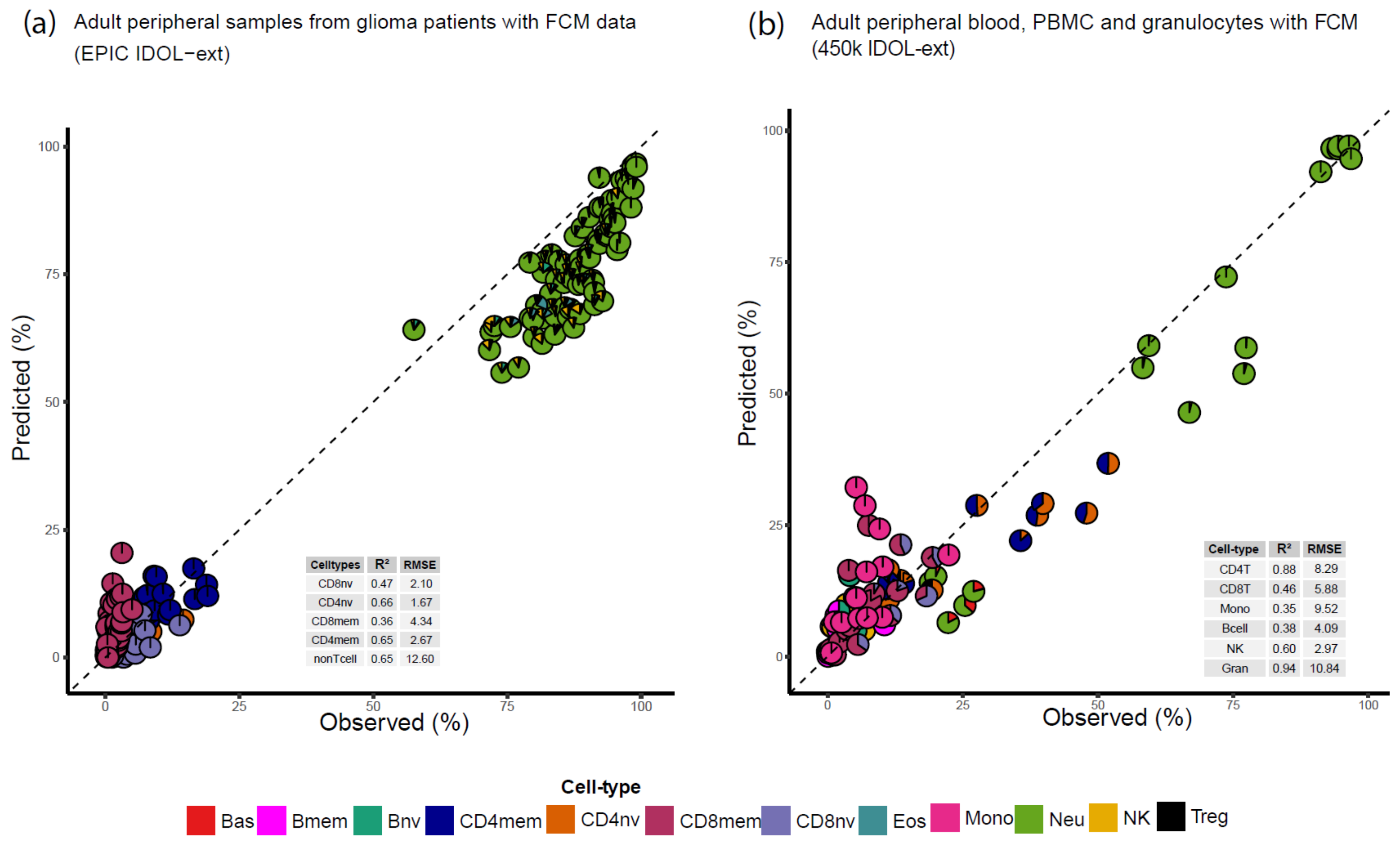

The area of each pieplot corresponds to the estimated proportion of the cell types within each group. (a) (EPIC data) includes information from the T-cell memory and naïve subtypes only. The unmeasured cells are summed as non-Tcells. (b) (450k) Gran (Granulocytes) corresponds to the sum of Neu-neutrophils, Eos-eosinophils, and Bas-basophils. CD4Tcorresponds to the sum of CD4+ T cells naïve, memory and Treg. CD8T corresponds to the sum of CD8+ T cells naïve and memory. Bcell to the sum of the naïve and memory. Information in panel B was retrieved from the supplementary materials from Reinius et al. 2012 for whole blood samples, peripheral blood mononuclear cells, and granulocytes. Data are available at GSE180683 (a) and GSE35069 (b). See Supplementary Table 1 for the abbreviations. Source data are provided as a Source Data file.

Supplementary Fig. 7. Exploratory analysis applying the libraries to umbilical cord blood datasets.

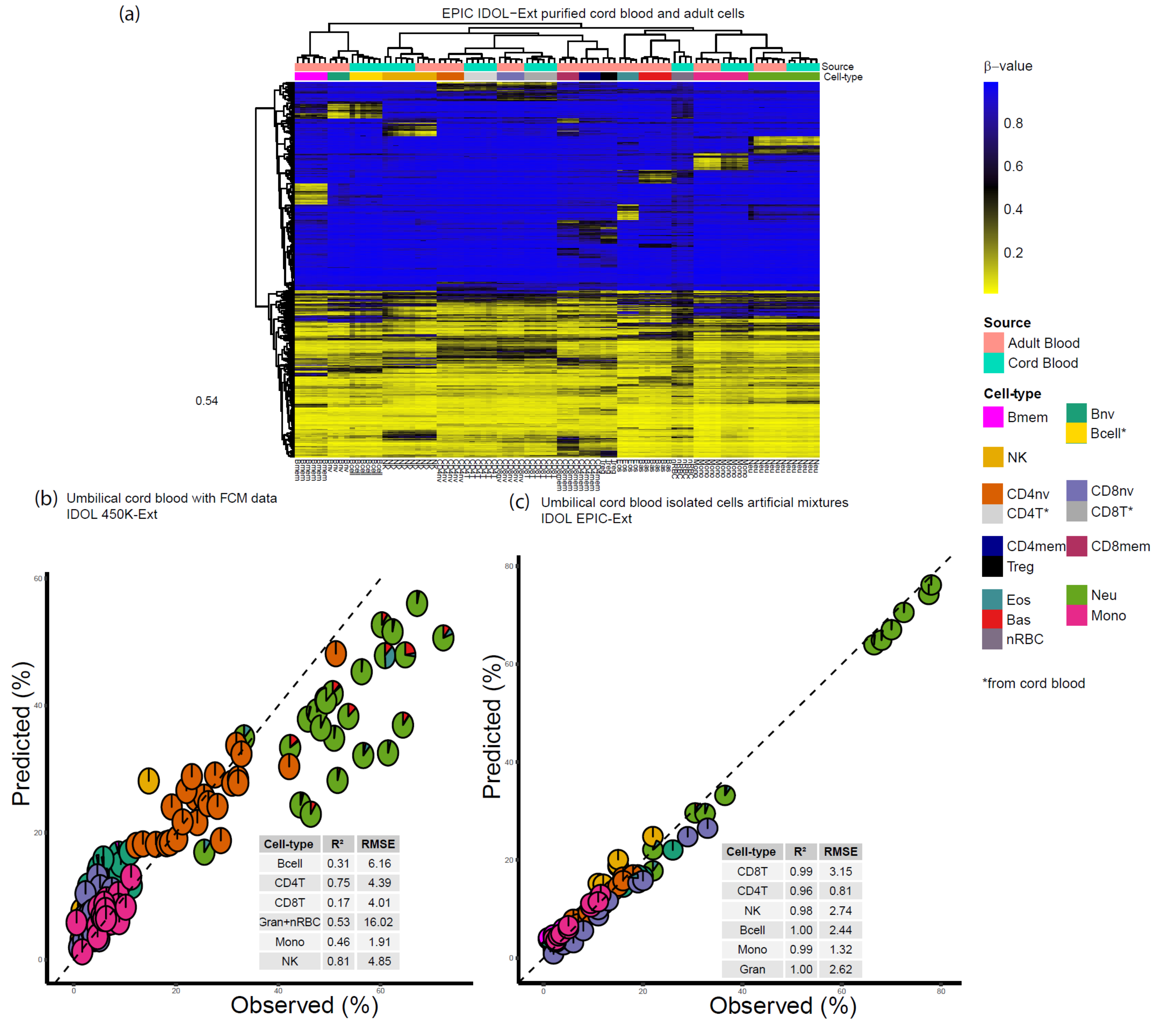

Cell-type	R²	RMSE
Bcell	0.31	6.16
CD4T	0.75	4.39
CD8T	0.17	4.01
Gran+nRBC	0.53	16.02
Mono	0.46	1.91
NK	0.81	4.85

Cell-type	R²	RMSE
CD8T	0.99	3.15
CD4T	0.96	0.81
NK	0.98	2.74
Bcell	1.00	2.44
Mono	0.99	1.32
Gran	1.00	2.62

(a) Heatmap of the EPIC IDOL-Ext probes and purified cell types in adult and umbilical cord blood including nRBC (nucleated red blood cells). (b) (450K IDOL-Ext): contains FCM information from umbilical cord blood samples. (c) (EPIC IDOL-Ext) corresponds to artificial mixtures using cells isolated from umbilical cord blood. The area of each pieplot corresponds to the estimated proportion of the cell-types within each group. In (b) (450k IDOL-ext) Gran (Granulocytes) corresponds to the sum of Neu-neutrophils, Eos-eosinophils, and Bas-basophils. CD4Tcorresponds to the sum of CD4+ T cells naïve, memory and Treg. CD8T corresponds to the sum of CD8+ T cells naïve and memory. Bcell to the sum of the naïve and memory. Data are available from GSE68456 and GSE180970). See Supplementary Table 1 for the abbreviations.Source data are provided as a Source Data file.

Supplementary Fig. 8. eForge 2.0 enrichment for differentially methylated regions ENCODE H3K4me1 (primed enhancers)

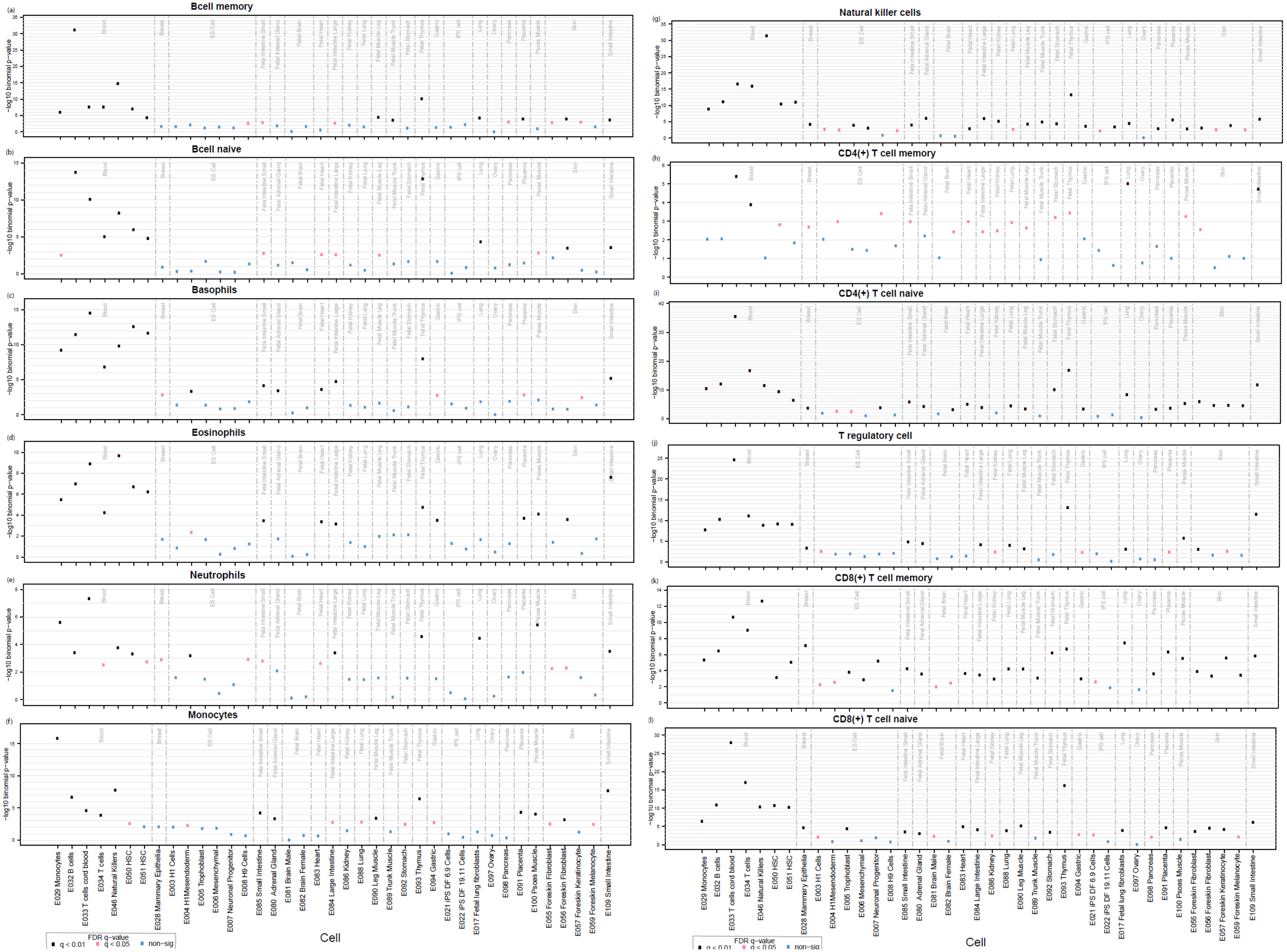

Data is derived from Supplementary Data 1 using the eForge tool. Source data are provided as a Source Data file.

Supplementary Table 6. Baseline characteristics of the samples included in the application datasets from GEO and ArrayExpress

Dataset (Platform)	N	Mean Age (SD)	N Male (%)	N European (%)	N African (%)	N East Asian (%)	Sample	Data Source
**Multiple Sclerosis (450k)**								
Case	13	44.53 (15.37)*	3 (23.08)*	13 (100)*	0 (0)*	0 (0)*	whole blood	GSE88824
Control	14	40.47 (11.91)*	5 (35.71)*	14 (100)*	0 (0)*	0 (0)*	whole blood	GSE88824
**Rheumatoid Arthritis (450k)**								
Case	354	51.15 (12.05)	101 (28.5)	354 (100)*	0 (0)*	0 (0)*	peripheral blood leukocytes	GSE42861
Control	335	52.76 (11.48)	96 (28.7)	335 (100)*	0 (0)*	0 (0)*	peripheral blood leukocytes	GSE42861
**Breast Cancer Treatment (EPIC)**								
Radiation-therapy	74	57.38 (9.24)	0 (0)	66 (89.19)*	2 (2.7)*	6 (8.11)*	peripheral blood	GSE140038
Radiation-therapy and chemotherapy	70	56.34 (11.15)	0 (0)	53 (75.71)*	4 (5.71)*	13 (18.57)*	peripheral blood	GSE140038
**COVID-19 (EPIC)**								
Healthy	6	55.8 (6.15)	0 (0)*	6 (100)*	0 (0)*	0 (0)*	peripheral blood	GSE161678
No Remission	2 (3 samples)	64.67 (13.28)	0 (0)*	2 (100)*	0 (0)*	0 (0)*	peripheral blood	GSE161678
Remission	4 (15 samples)	65.73 (12.79)	1 (25)*	4 (100)*	0 (0)*	0 (0)*	peripheral blood	GSE161678
**Twin (450k)**								
Monozygotic twins	852	18 (0)	438 (51.4)	829 (97.3)*	23 (2.7)*	0 (0)*	whole blood	GSE105018
Dizygotic twins	612	18 (0)	312 (50.1)	597 (97.55)*	15 (2.45)*	0 (0)*	whole blood	GSE105018
**Aging (450k+EPIC)**								
Newborn	141	0 (0)	62 (44.0)	117 (82.98)*	24 (17.02)*	0 (0)*	cord blood	E-MTAB-7069, GSE85042, GSE103189, GSE104778
0-5	71	2.73 (1.87)	6 (8.5)	68 (95.77)*	3 (4.23)*	0 (0)*	peripheral blood	E-MTAB-7069, GSE62219
5-18	95	15.71 (2.33)	47 (49.5)	95 (100)*	0 (0)*	0 (0)*	peripheral blood	E-MTAB-7069, GSE87571
18-65	1049	49.93 (12.43)	317 (30.2)	1045 (99.62)*	3 (0.29)	1 (0.1)*	peripheral blood	E-MTAB-7309, GSE87571, GSE121633
>65	1148	75.75 (7.07)	408 (35.5)	1148 (100)*	0 (0)*	0 (0)*	peripheral blood	E-MTAB-7309, GSE87571, GSE121633
Total	4852							

* Inferred age, sex, and "ethnicity" (general genetic ancestry markers) based on methylation using *SeSAMe*

Supplementary Fig. 9. Predicted immune cell proportions in whole blood samples between multiple sclerosis cases (n=13) and normal controls (n=14) (450k)

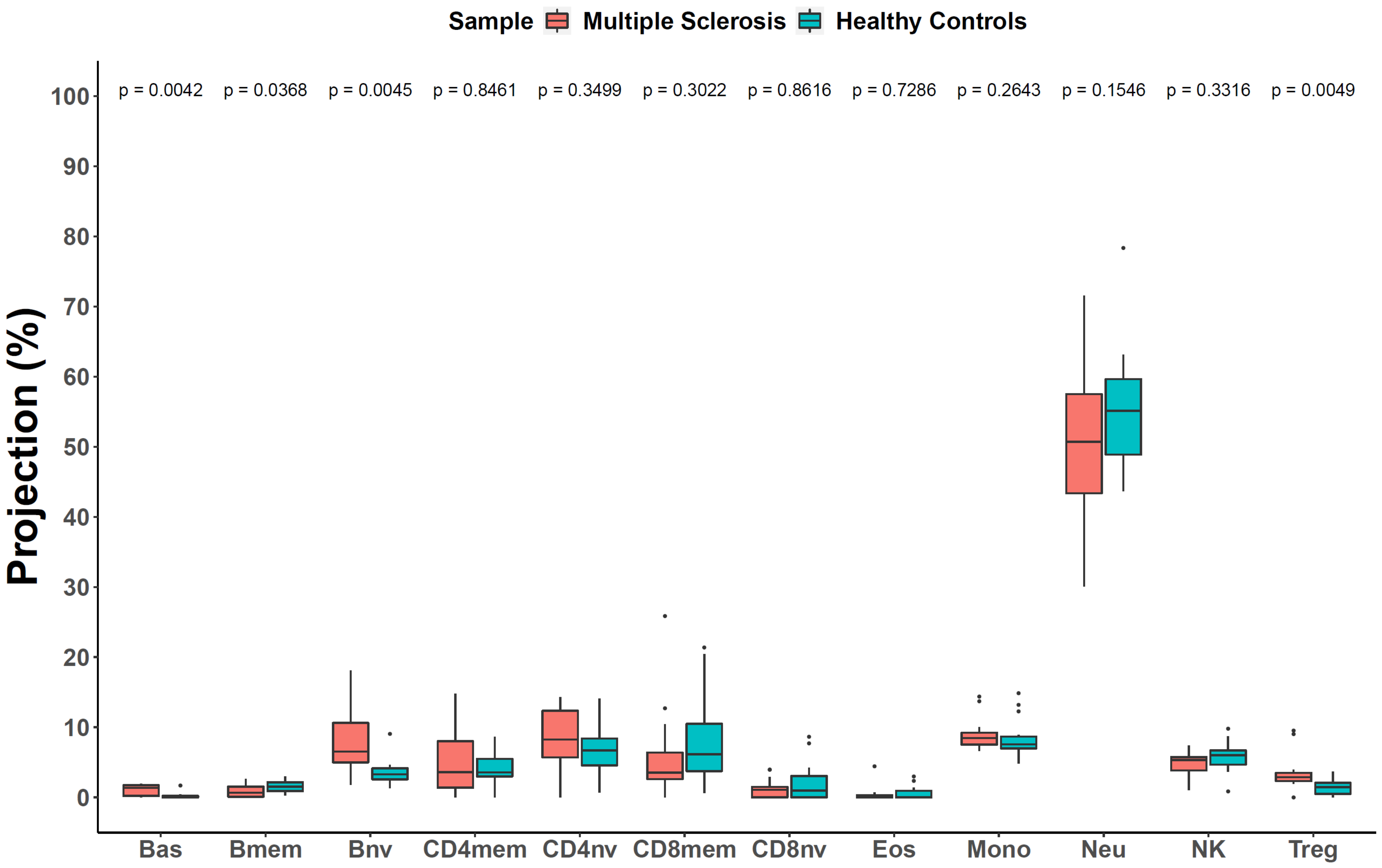

The boxplots include the following information: (1) The box shows the interquartile range (IQR), (2) the whiskers show the inner fences (1.5 × IQR out of the box), (3) the bolded line shows the median of the data. A two-sided Wilcoxon test was used to derive the p-values. P-values are not corrected for multiple comparisons. Data are derived from GSE88824. See Supplementary Table 1 for the abbreviations. Source data are provided as a Source Data file.

Supplementary Fig. 10. Predicted immune cell proportions in peripheral blood leukocyte samples between rheumatoid arthritis cases (n=354) and normal controls (n=335) (450k)

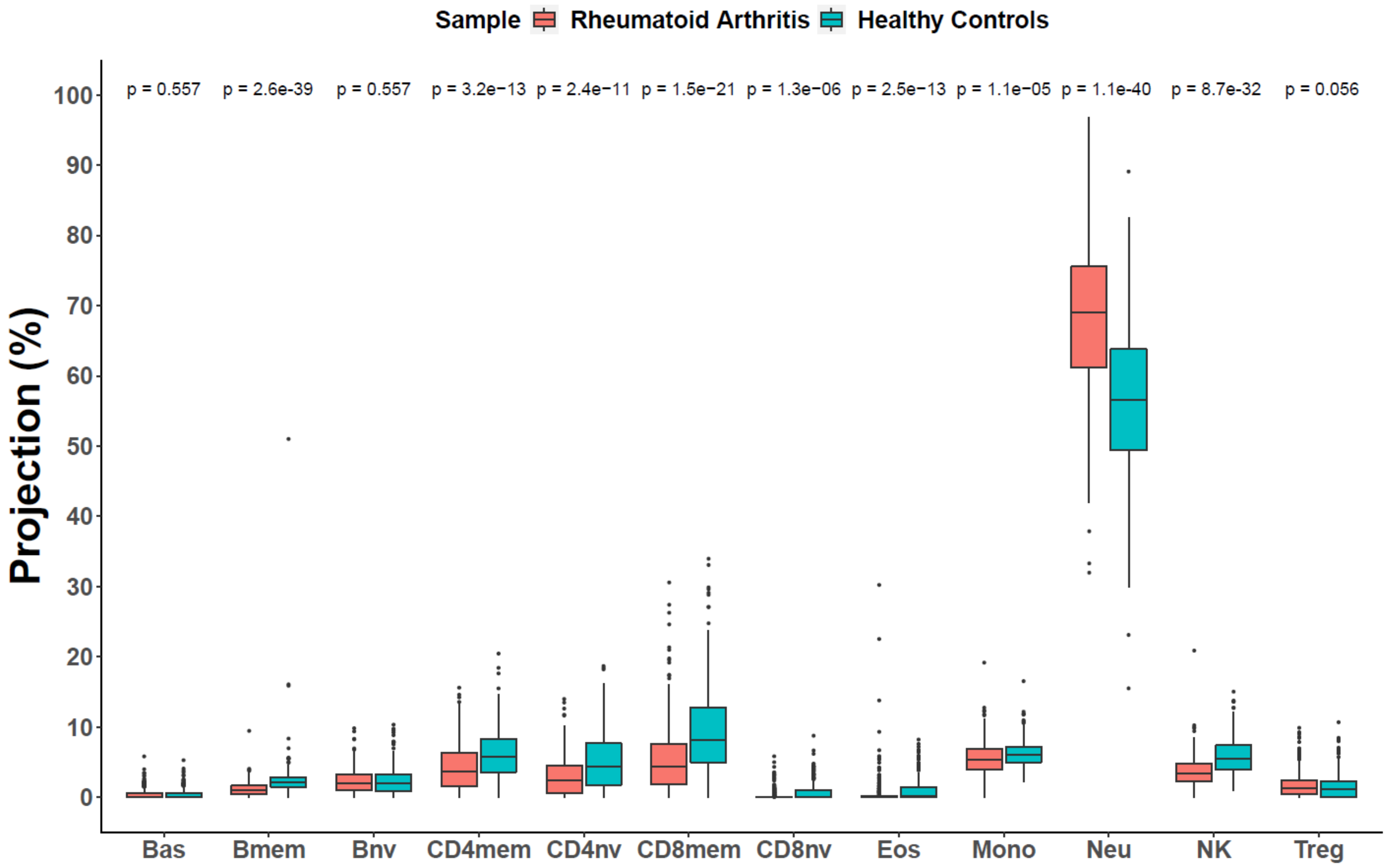

The boxplots include the following information: (1) The box shows the interquartile range (IQR), (2) the whiskers show the inner fences (1.5 × IQR out of the box), (3) the bolded line shows the median of the data. A two-sided Wilcoxon test was used to derive the p-values. P-values are not corrected for multiple comparisons. Data are derived from GSE42861. See Supplementary Table 1 for the abbreviations. Source data are provided as a Source Data file.

Supplementary Fig. 11. Predicted immune cell proportions in peripheral blood samples from early breast cancer patients before and after receiving radiation therapy only (a n=74) and radiation therapy plus chemotherapy (b, n=70) (EPIC)

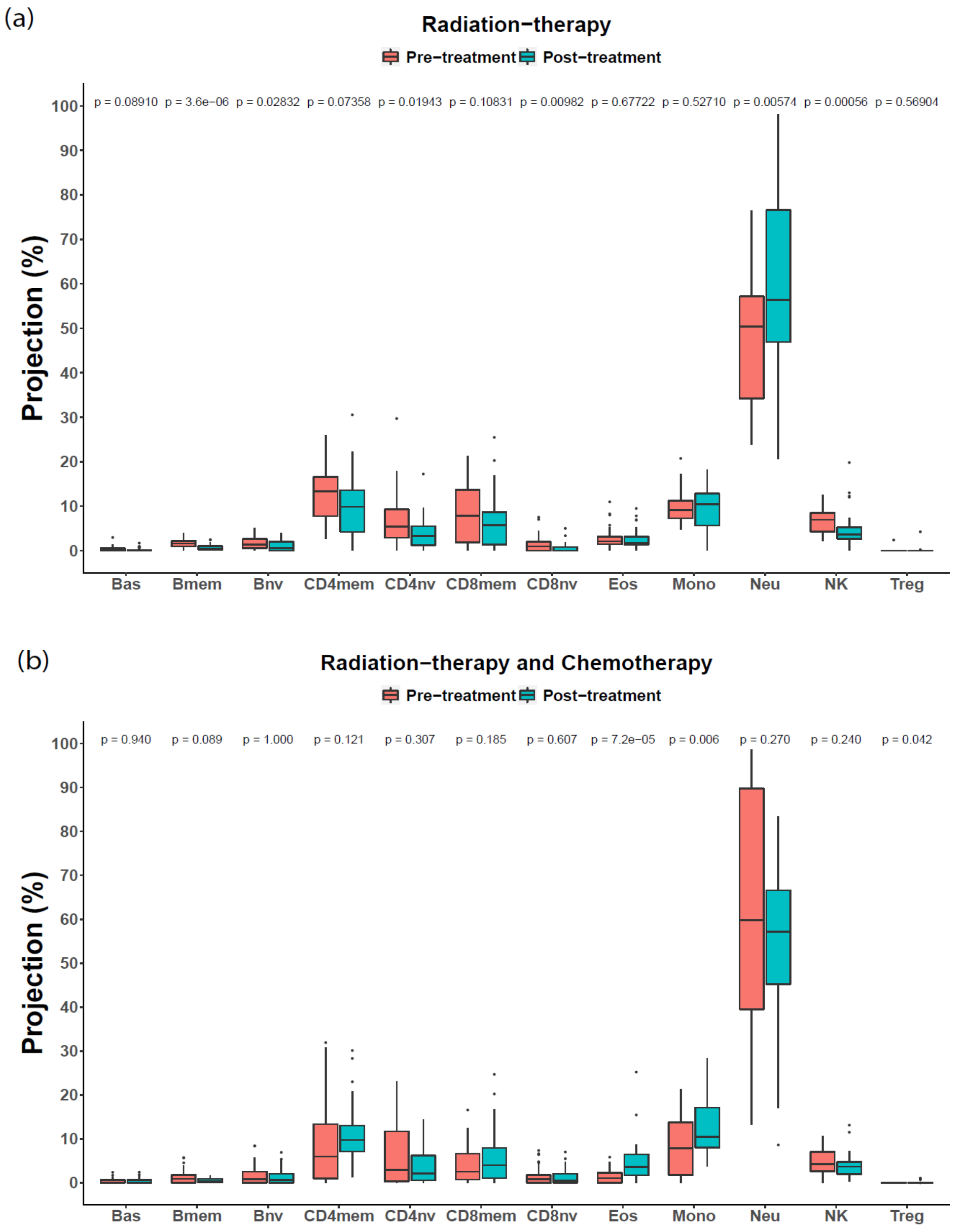

The boxplots include the following information: (1) The box shows the interquartile range (IQR), (2) the whiskers show the inner fences (1.5 × IQR out of the box), (3) the bolded line shows the median of the data. A two-sided Wilcoxon test was used to derive the p-values. P-values are not corrected for multiple comparisons. Data are derived from GSE140038. See Supplementary Table 1 for the abbreviations.

Supplementary Fig. 12. Changes in estimated immune cell proportions between subjects with COVID-19 infection with and without remission compared to healthy subjects (EPIC)

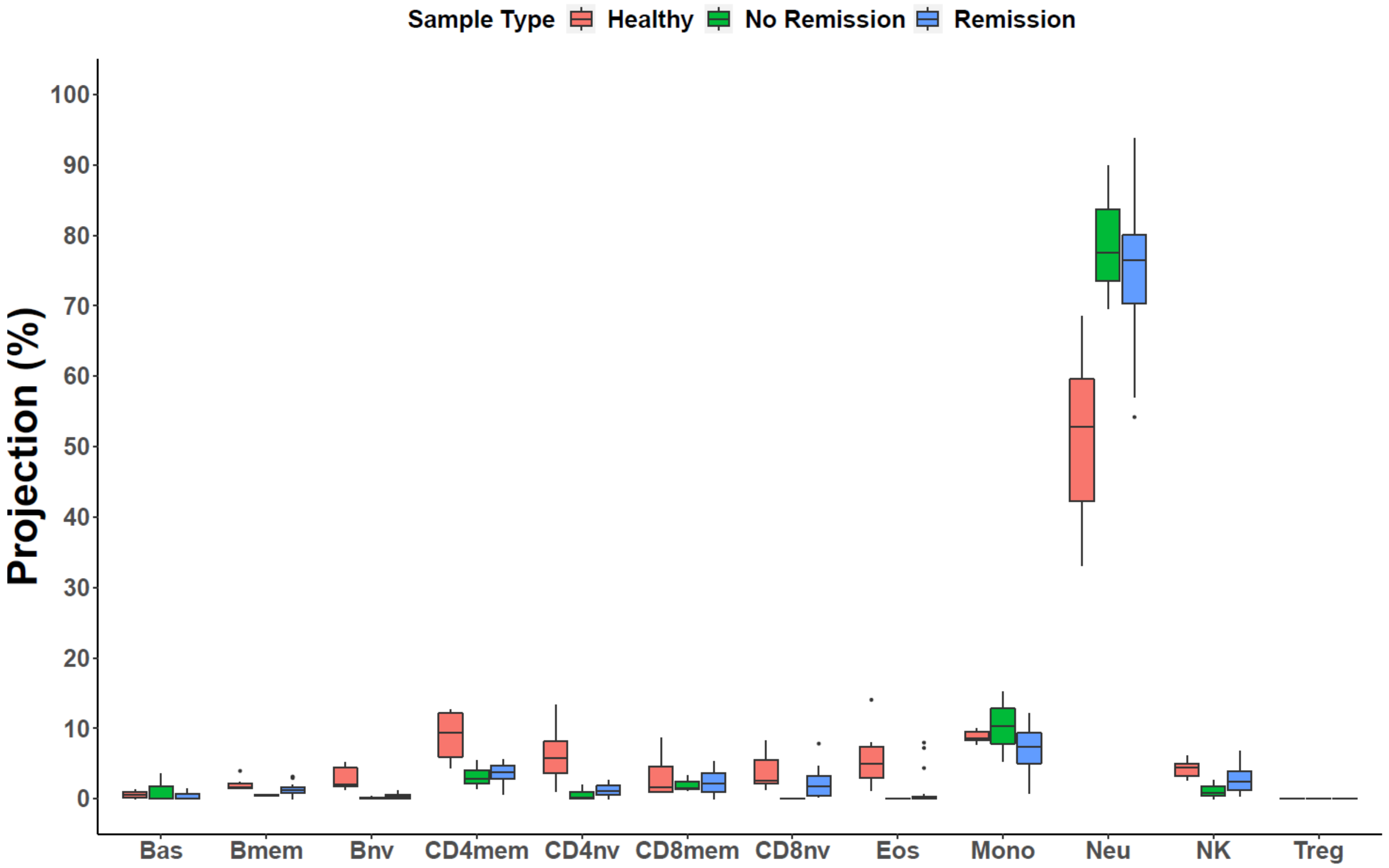

The boxplots include the following information: (1) The box shows the interquartile range (IQR), (2) the whiskers show the inner fences (1.5 × IQR out of the box), (3) the bolded line shows the median of the data. Given the limited number of samples, P-values were not calculated. Data are derived from GSE161678. See Supplementary Table 1 for the abbreviations. Source data are provided as a Source Data file.

Supplementary Fig. 13. Differences of predicted immune cell proportions in whole blood samples between pairs of twins in monozygotic twins (n=852) and dizygotic twins (n=612) (450k)

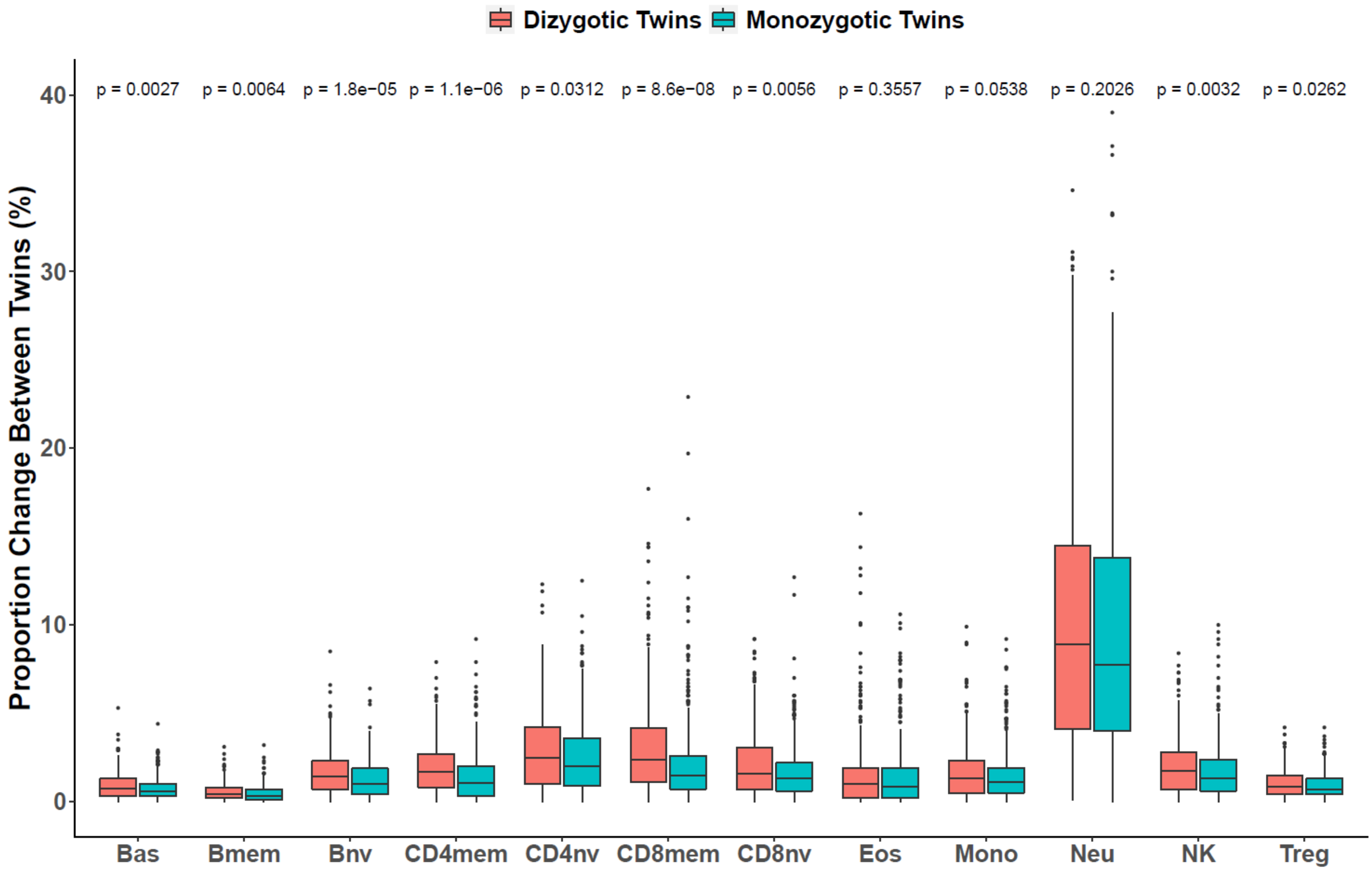

The boxplots include the following information: (1) The box shows the interquartile range (IQR), (2) the whiskers show the inner fences (1.5 × IQR out of the box), (3) the bolded line shows the median of the data. A two-sided Wilcoxon test was used to derive the p-values. P-values are not corrected for multiple comparisons. Data are derived from GSE105018. See Supplementary Table 1 for the abbreviations.Source data are provided as a Source Data file.

Supplementary Fig. 14. Trajectories of several cell subpopulation ratios across different ages (in years) using publicly available datasets (n=2504)

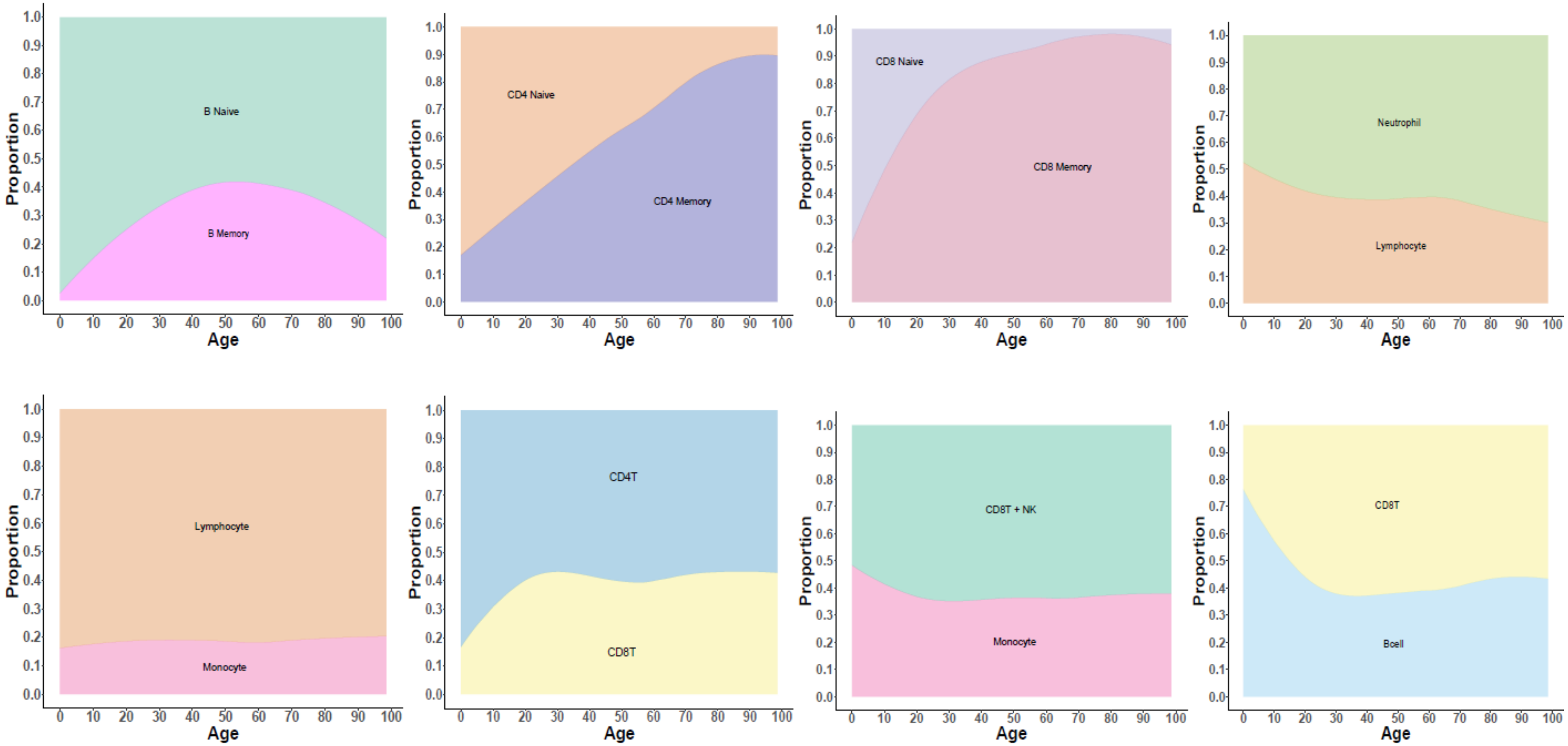

The estimated cells' proportion was averaged and smoothed to represent the proportion for the compartment and the ratio between the subpopulations for specific ages (in years). The subjects represent "healthy controls"; however, it is unknown if specific subjects had any comorbidity at the time of the sample array (For a granular representation of the data, see Supplementary Fig. S15). See Supplementary Table 7 for the sources of the data. See Supplementary Table 1 for the abbreviations. Source data are provided as a Source Data file.

Supplementary Fig. 15. Changes in predicted immune cell proportions with aging using samples from ages zero to greater than 90 years (n=2504) (450k and EPIC)

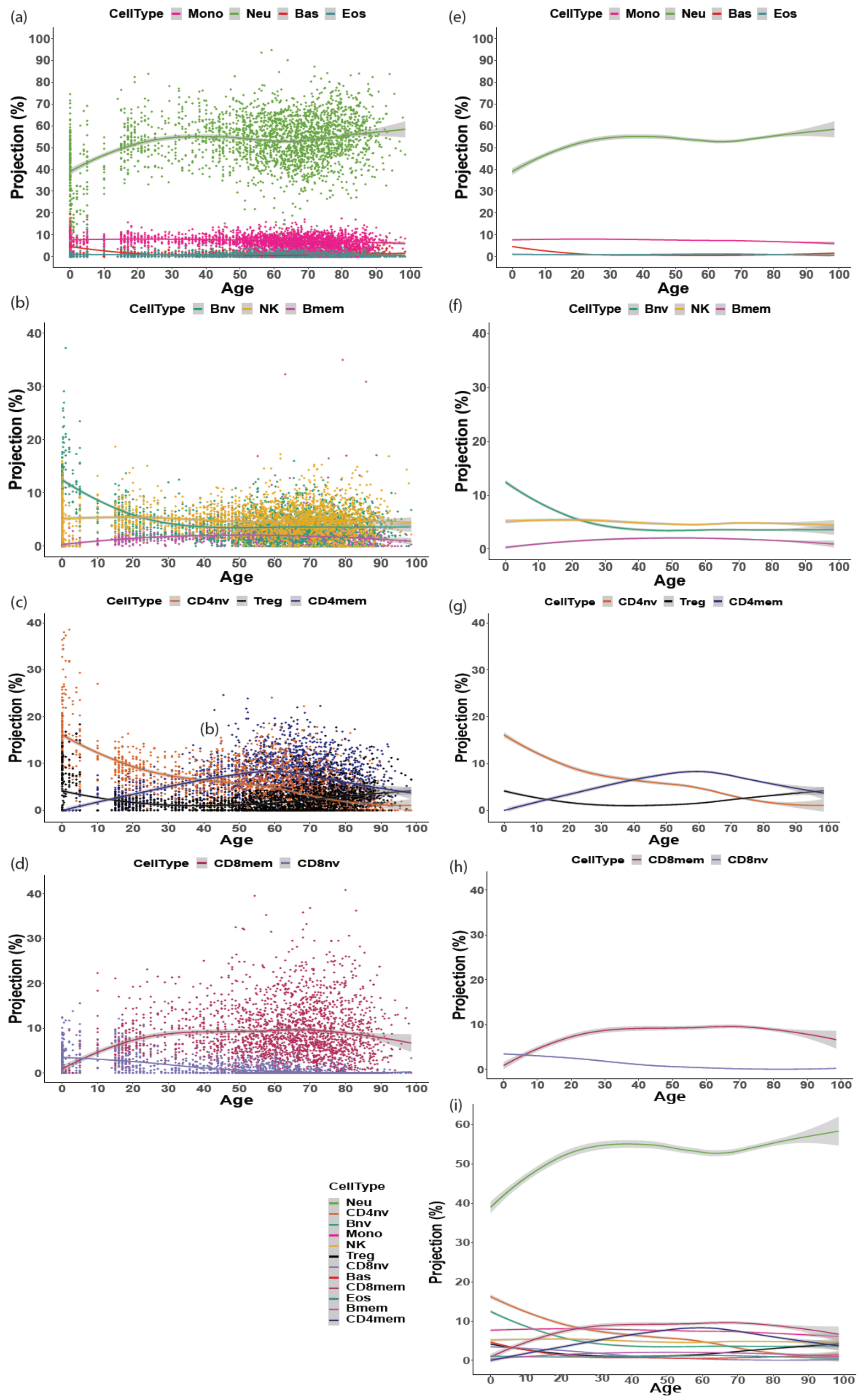

Panels a-d show the granular projections per cell type. Panels e-i show the cubic spline projections (solid line colored by cell-type) and 95% CI (gray shade around the lines). See Supplementary Table 7 for the sources of the data. See Supplementary Table 1 for the abbreviations. Source data are provided as a Source Data file.

Supplementary Fig. 16. Longitudinal changes of predicted immune cell proportions between 3 months to 5 years after birth in human blood leukocytes of 10 healthy girls

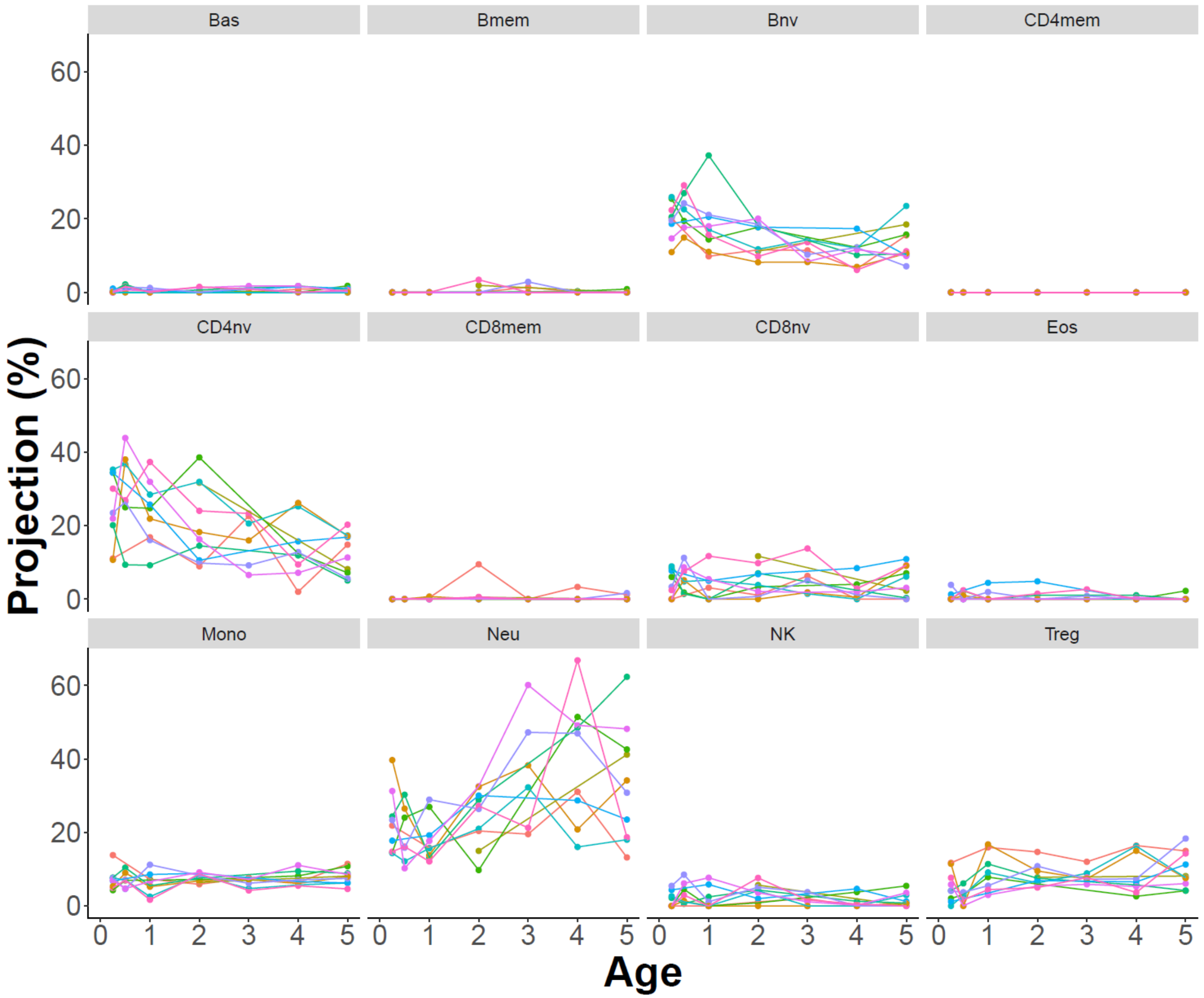

Each color represents one subject followed over time (7-time points). Data is derived from GSE62219. See Supplementary Table 1 for the abbreviations. Source data are provided as a Source Data file.

Supplementary Fig. 17. Bland Altman plots comparing the estimation using constrained projection/quadratic programming (CP/QP) versus the true values using the EPIC IDOL-Ext and the 450k IDOL-Ext libraries

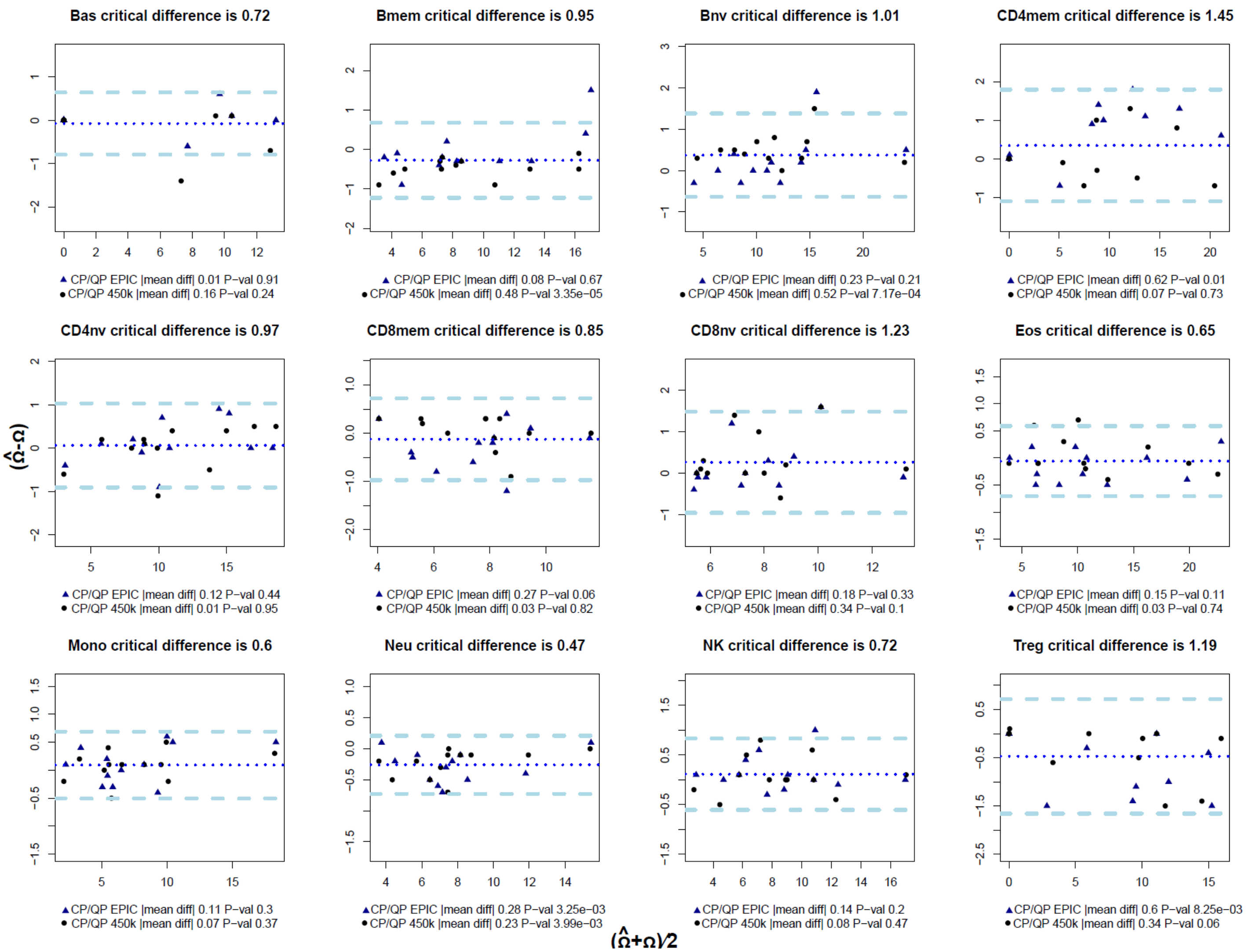

The x-axes are the average between the estimated ($\widehat{\Omega}$) versus the true values (Ω) per cell-type, and the y-axes the differences between the estimated ($\widehat{\Omega}$) versus the true values (Ω) per cell type. Paired t-tests were calculated per library and cell type. Absolute mean differences (|mean diff|) and P values are reported. The darker blue dotted line represents the mean of the differences, and the lighter blue dashed lines 1 SD of the 95% CI limit of agreement. Data can be derived from GSE167998. Source data are provided as a Source Data file.

Supplementary Fig. 18. Bland Altman plots comparing three different statistical deconvolution methods (CIBERSORT- CBS, constrained projection/quadratic programming-CP/QP, robust partial correlations-RPC) estimates versus the true values using the EPIC IDOL-Ext library

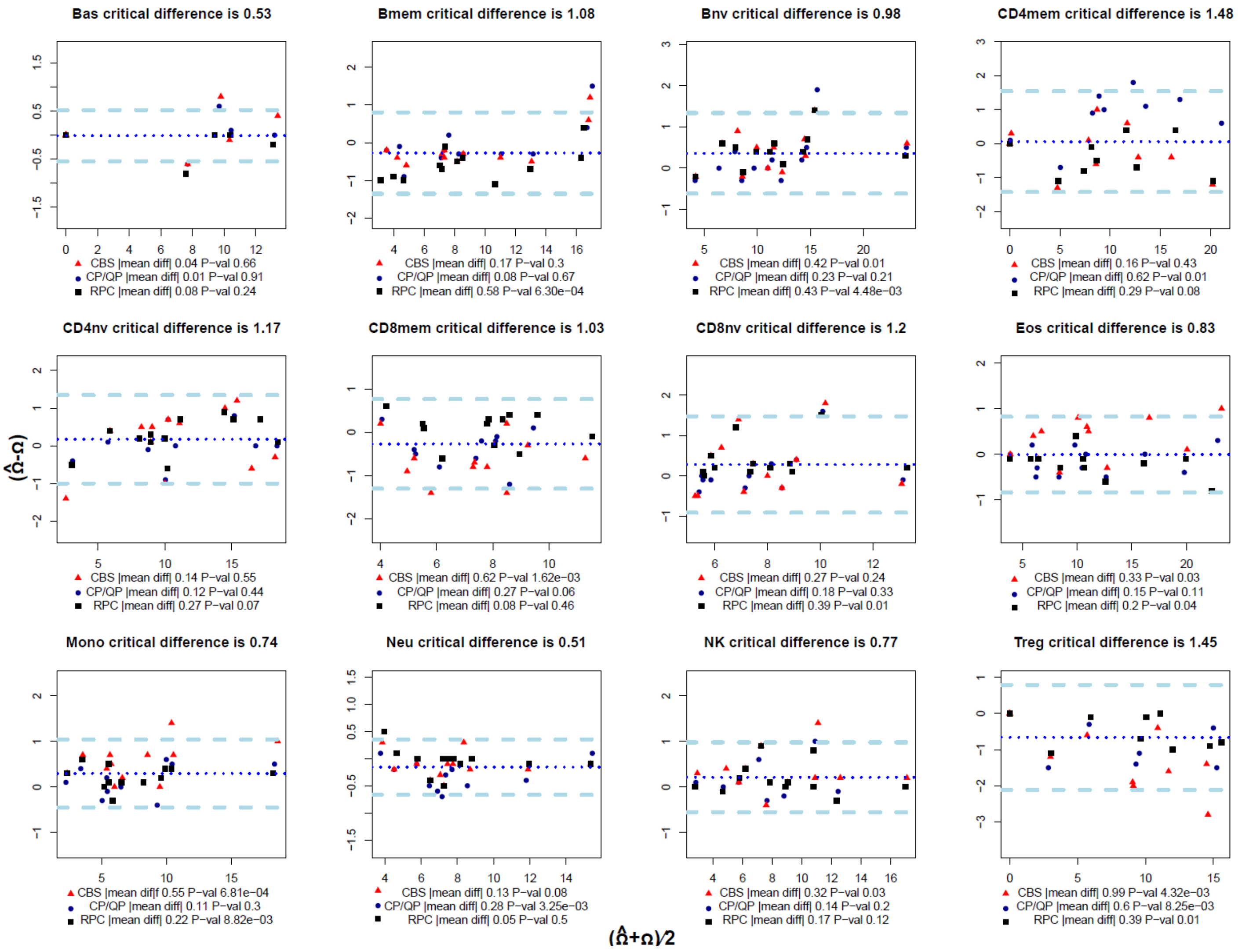

The x-axes are the average between the estimated ($\hat{\Omega}$) versus the true values (Ω) per cell-type, and the y-axes the differences between the estimated ($\hat{\Omega}$) versus the true values (Ω) per cell type. Paired t-tests were calculated per library and cell type. Absolute mean differences (|mean diff|) and P values are reported. The darker blue dotted line represents the mean of the differences, and the lighter blue dashed lines 1 SD of the 95% CI limit of agreement. Data can be derived from GSE167998. Source data are provided as a Source Data file.
