# Supplementary material for: Enhanced cell deconvolution of peripheral blood using DNA methylation for high-resolution immune profiling
Source: Nat Commun. 2022 Feb 9;13:761. doi: 10.1038/s41467-021-27864-7 (PMC8828780; doi:10.1038/s41467-021-27864-7)
Supplement: Supplementary file 4 — List of Supplementary Information [file 41467_2021_27864_MOESM4_ESM.docx]

**Data Files**

Supplementary Data File 1. IDOL Optimized probes in the EPIC IDOL-Ext (n=1200)

Supplementary Data File 2. IDOL Optimized probes in the 450k IDOL-Ext (n=1500)

Supplementary Data File 3. Automatic selected probes using pickCompProbes in minfi (n=1200)

Supplementary Data File 4. Gene Enrichment Analyses using missMethyl and the MSigDB (GSEA) curated database v7.2
