# Supplementary material for: Enhanced cell deconvolution of peripheral blood using DNA methylation for high-resolution immune profiling
Source: Nat Commun. 2022 Feb 9;13:761. doi: 10.1038/s41467-021-27864-7 (PMC8828780; doi:10.1038/s41467-021-27864-7)
Supplement: Supplementary file 9 — Reporting Summary [file 41467_2021_27864_MOESM9_ESM.pdf]

nature research

Corresponding author(s): Lucas A Salas
Last updated by author(s): Nov 15, 2021

Reporting Summary

Nature Research wishes to improve the reproducibility of the work that we publish. This form provides structure for consistency and transparency in reporting. For further information on Nature Research policies, see our Editorial Policies and the Editorial Policy Checklist.

Statistics

For all statistical analyses, confirm that the following items are present in the figure legend, table legend, main text, or Methods section.

n/a	Confirmed	
☐	☒	The exact sample size (*n*) for each experimental group/condition, given as a discrete number and unit of measurement
☐	☒	A statement on whether measurements were taken from distinct samples or whether the same sample was measured repeatedly
☐	☒	The statistical test(s) used AND whether they are one- or two-sided<br>*Only common tests should be described solely by name; describe more complex techniques in the Methods section.*
☐	☒	A description of all covariates tested
☐	☒	A description of any assumptions or corrections, such as tests of normality and adjustment for multiple comparisons
☐	☒	A full description of the statistical parameters including central tendency (e.g. means) or other basic estimates (e.g. regression coefficient) AND variation (e.g. standard deviation) or associated estimates of uncertainty (e.g. confidence intervals)
☐	☒	For null hypothesis testing, the test statistic (e.g. *F*, *t*, *r*) with confidence intervals, effect sizes, degrees of freedom and *P* value noted<br>*Give P values as exact values whenever suitable.*
☒	☐	For Bayesian analysis, information on the choice of priors and Markov chain Monte Carlo settings
☒	☐	For hierarchical and complex designs, identification of the appropriate level for tests and full reporting of outcomes
☒	☐	Estimates of effect sizes (e.g. Cohen's *d*, Pearson's *r*), indicating how they were calculated

Our web collection on statistics for biologists contains articles on many of the points above.

Software and code

Policy information about availability of computer code

Data collection	Data from publicly available sources were obtained using R v4.1.0 using GEOquery and ArrayExpress
Data analysis	The methylation array raw idat files were pre-processed using minfi, EnMIX, and SeSaMe for quality control using R v.4.0.2 and 4.1.0 Data was analyzed. Code is available on github https://github.com/immunomethylomics/FlowSorted.BloodExtended.EPIC and Zenodo doi: 10.5281/zenodo.5338513

For manuscripts utilizing custom algorithms or software that are central to the research but not yet described in published literature, software must be made available to editors and reviewers. We strongly encourage code deposition in a community repository (e.g. GitHub). See the Nature Research guidelines for submitting code & software for further information.

Data

Policy information about availability of data

All manuscripts must include a data availability statement. This statement should provide the following information, where applicable:

- Accession codes, unique identifiers, or web links for publicly available datasets
- A list of figures that have associated raw data
- A description of any restrictions on data availability

The main source dataset (12 cell types, testing and training artificial mixtures) is hosted on GSE167998. The Superseries GSE181034 cis composed of the following series GSE180683 (glioma samples, validations for T cell memory subsets), GSE180970 (umbilical cord artificial mixtures), GSE182379 (independent validation 12 cell-types artificial mixtures). All the datasets will be public upon the manuscript acceptance. Additional datasets analyzed in this manuscript are publicly available in GEO and ArrayExpress with accession numbers: GSE110554, GSE77797, GSE110530, GSE35069, GSE68456, GSE88824, GSE42861, GSE140038, GSE161778, GSE105018, E-MTAB-7069, GSE85042, GSE103189, GSE104778, GSE62219, GSE87571, E-MTAB-7309, GSE87571, GSE12163.The code used for this manuscript has been deposited in Zenodo doi: 10.5281/zenodo.5338513. All the source data has been added as source data file to the manuscript.

Field-specific reporting

Please select the one below that is the best fit for your research. If you are not sure, read the appropriate sections before making your selection.

☒ Life sciences ☐ Behavioural & social sciences ☐ Ecological, evolutionary & environmental sciences

For a reference copy of the document with all sections, see nature.com/documents/nr-reporting-summary-flat.pdf

Life sciences study design

All studies must disclose on these points even when the disclosure is negative.

Sample size	No formal sample size was calculated. The biological signals should be consistent for the isolated cell-types in the analysis.
Data exclusions	Samples with lower levels of purity (<85% in the DNA methylation purity or flow cytometry estimates) were excluded from the library.
Replication	External datasets with cell-type information were used for external validation. 12 independent artificial mixtures (samples not included in the discovery dataset) were used to replicate the results of the library.
Randomization	Samples were not randomized. Internal validity included random generation of the proportions used for the training, testing and validation.
Blinding	No blinding was feasible for this analysis, samples were assigned based on cell isolation, and proportions were known.

Reporting for specific materials, systems and methods

We require information from authors about some types of materials, experimental systems and methods used in many studies. Here, indicate whether each material, system or method listed is relevant to your study. If you are not sure if a list item applies to your research, read the appropriate section before selecting a response.

Materials & experimental systems

n/a	Involved in the study
☒	☐ Antibodies
☒	☐ Eukaryotic cell lines
☒	☐ Palaeontology and archaeology
☒	☐ Animals and other organisms
☐	☒ Human research participants
☒	☐ Clinical data
☒	☐ Dual use research of concern

Methods

n/a	Involved in the study
☒	☐ ChIP-seq
☒	☐ Flow cytometry
☒	☐ MRI-based neuroimaging

Human research participants

Policy information about studies involving human research participants

Population characteristics	Cells were isolated from 41 males and 15 females, all anonymous healthy donors. The donors had a mean age of 32.2 years (sd= 12.2, range 19–58 years) and an average weight of 85.1 kg (range 57–136 Kg). Donors identified themselves from multiple ethnicities, including mixed ethnicities, and were categorized broadly into four groups (African-Americans, East-Asian, Indo-European, multiple/admixed). They were negative for Human Immunodeficiency Virus-HIV, Hepatitis B Virus-HBV, and Hepatitis C Virus-HCV. Women were not pregnant at the time of sample collection, and samples were collected from donors with no history of heart, lung, kidney disease, asthma, blood disorders, autoimmune disorders, cancer, or diabetes. All donors provided written informed consent before donation. The data discussed in this publication have been deposited in NCBI's Gene Expression Omnibus (Salas et al., 2021) and are accessible through GEO Series accession number GSE167998 (https://www.ncbi.nlm.nih.gov/geo/query/acc.cgi?acc=GSE167998). Isolation protocols are available through the commercial websites of AllCells, StemExpress, and STEMCELL Technologies. In brief, cells were selected using immunomagnetic labeling through the vendors' specific protocols (see, Supplementary Table 1 for details). Additional publicly available datasets are described in detail in supplementary tables.
Recruitment	Samples are from commercial vendors, they recruited volunteers for cell isolation.
Ethics oversight	All the subjects participation was voluntary. Subjects are consented, and they receive a compensation for their participation from the companies providing the samples, not from the researchers.

Note that full information on the approval of the study protocol must also be provided in the manuscript.
