# Supplementary material for: Enhanced cell deconvolution of peripheral blood using DNA methylation for high-resolution immune profiling
Source: Nat Commun. 2022 Feb 9;13:761. doi: 10.1038/s41467-021-27864-7 (PMC8828780; doi:10.1038/s41467-021-27864-7)
Supplement: Supplementary file 3 — Editorial Assessment Report [file 41467_2021_27864_MOESM3_ESM.pdf]

Editorial Assessment Report

Dear Dr. Salas,

Thank you again for choosing to submit your manuscript using the Guided Open Access pilot at the Nature Portfolio. As part of this process, our editorial team has considered your paper for three of our journals with strong interest in publishing in your field: *Nature Methods*, *Nature Communications*, and *Communications Biology*.

Your manuscript entitled "Enhanced cell deconvolution of peripheral blood using DNA methylation for high-resolution immune profiling" has now been reviewed by 2 experts in computational biology, deconvolution approaches, and methylomic analyses, whose comments are included in the attached Editorial Assessment Report. As part of the Guided Open Access pilot, editors from all 3 journals have discussed the reviewer reports and the manuscript's suitability for our journals. After careful evaluation, our editorial recommendation is to revise the manuscript and submit back through the Guided Open Access submission portal for consideration at *Communications Biology* or *Nature Communications*. Provided the revisions satisfy all technical and editorial concerns, *Communications Biology* is especially interested in publishing your manuscript. Please see details in the attached Editorial Assessment Report.

In brief, for publication in *Communications Biology*, we would require you to validate this approach in an independent dataset containing all 12 cell types, and address the referee concerns regarding methodological advance, data availability, and tissue-specific methylation patterns. For consideration in *Nature Communications*, the editors would request all of these edits, as well as the addition of subject demographic information and a discussion of how the composition of this cohort might impact the generalizability of your method to other datasets. You will find more details regarding the required revisions in the Editorial Assessment Report below.

Please note that the Editorial Assessment Report is a standalone document that contains an editorial evaluation, recommendation and portable peer advice to help you navigate and interpret the reviewers' reports. It also provides guidance for adhering to best practice with regard to transparency and reproducibility, for example on the issue of sharing data. We have also included information about data accessibility and reproducibility, which we hope you find useful.

Should you have any questions about the recommended journals or would like advice on the revisions, you can contact me directly and I will be happy to assist. We look forward to receiving the revised version of your manuscript.

Sincerely,
George Inglis
--
George Inglis
Associate Editor
Communications Biology

On behalf of the Guided OA editorial team

Contents of this Report

- **Manuscript overview**: details about your manuscript and the editorial team.
- **Manuscript assessment**: personalised recommendation from the editors.
- **About the editorial process**: an overview of the Guided Open Access process.
- **Annotated reviewer comments**: the referee reports with comments from the editors.
- **Open research evaluation**: advice for adhering to best reproducibility practices.

Manuscript overview

Manuscript number	Submission date	Decision date
GUIDEDOA-21-00075	20 March 2021	14 May 2021

**Title**	Enhanced cell deconvolution of peripheral blood using DNA methylation for high-resolution immune profiling	**Corresponding author**	Lucas Salas<br>ORCID: 0000-0002-2279-4097
**Preprint information**	https://www.biorxiv.org/content/10.1101/2021.04.11.439377v1	**Peer review type**	Single-blind
**Editorial Assessment Team**	**Primary editor: George Inglis**<br>**Home Journal:** *Communications Biology*, ORCID: 0000-0002-9069-5242<br>**Editorial team members:**<br>Madhura Mukhopadhyay, *Nature Methods*, ORCID: 0000-0003-3907-3955<br>Doaa Megahed, *Nature Communications*		
**About your primary editor**	George received his PhD in Genetics and Molecular Biology from Emory University, where he studied mouse models of voltage-gated sodium channel dysfunction and epilepsy. He also has research experience in epigenomics and in vitro models of neuronal development. George joined the editorial team of Communications Biology in September 2020 and is based in the New York office.		

Manuscript assessment and recommendation	
**Editor's summary of the manuscript and overall assessment**	Here, the authors describe a pipeline to accurately determine relative proportions of 12 leukocyte types from heterogeneous blood samples. Briefly, they used FACS to isolate each of these 12 cell subtypes from an overarching cohort of 56 healthy donors, then identified subtype-specific DNA methylation patterns using the EPIC or 450k methylation arrays. They trained their algorithm to identify proportions of these subtypes by mixing known ratios of purified DNA from each cell, and proceed to validate its utility on multiple publicly available datasets. Their results suggest that this deconvolution approach is highly accurate in predicting cell type proportions from bulk datasets, regardless of whether the data was obtained from the EPIC or 450k array.<br><br>The editors jointly decided to send this manuscript out to review, given the comprehensive expansion of this panel to 12 cell types, analysis of its suitability to EPIC and 450k arrays, and validation on multiple datasets. While we appreciate that this paper described an improvement over reference #19 (deconvolution of 5 subtypes), the fact that a similar method had already been published limited the methodological advance and prohibited further consideration by *Nature Methods*.<br><br>The referees largely find the study to be well-designed and of value to the broader research community. However, Referee #2 raises some concerns regarding validation of the approach, given that no truly independent datasets incorporating all 12 cell types were utilized. Referee #1 also notes the need to further discuss the advantages and limitations of this method to existing alternatives, and additional analysis of tissue-specific DNA methylation sites.<br><br>In summary, a resubmission should (at a minimum) contain the following revisions:<br>(1) Validate the accuracy of this method in an independent dataset containing all 12 cell types, as suggested by Referee #2.<br>(2) Carefully qualify the advantages and limitations of the approach compared to other methods, as outlined by Referee #1.<br>(3) Utilize eFORGE or a similar approach to examine tissue-specific methylated elements, as suggested by Referee #1.<br>(4) Carefully proofread for grammatical errors and clarity, as emphasized by both reviewers.

Editorial assessment overview

Nature Methods	Not feasible to meet the editorial requirements	The technical and conceptual advance (compared to prior literature) represented by the method is not sufficient for publication in *Nature Methods*.
Nature Communications	Major revisions needed	All revisions requested by *Communications Biology* (see below), plus discussion of subject demographic information and potential generalizability of results would be required for publication.
Communications Biology	Some additional data needed, but generally minor revisions	As noted by the reviewers, further validation in an independent dataset and discussion of the advantages provided by this approach would be necessary for publication

**Editor's recommendation**	***Option 1*: Revise for consideration at Nature Communications**<br>A revision should address all reviewer concerns and provide the demographic information of all subjects used in this study. Furthermore, it would be necessary to discuss potential limitations, such as the demographic composition of the source cohort, and whether it would affect the generalizability of the method.<br><br>***Option 2*: Revise and submit to Communications Biology**<br>While many revisions are needed, these do not require any new experiments beyond validating the method in an independent dataset, and discussing limitations or advantages of the approach.<br><br>**Please state in your cover letter which journal you have revised for.**
**Next steps**	If you would like to follow our recommendation, when you are ready you can upload the revised manuscript, along with your point-by-point response to the reviewer's reports and editorial advice **here***.

About the editorial process

By selecting the Nature Portfolio Guided Open Access option, your manuscript was assessed for suitability in three of our titles that provide venues for publication of high-quality work across the spectrum of methods development research: *Nature Methods, Nature Communications*, and *Communications Biology*. For more information about Guided Open Access, please see here.

Collaborative editorial assessment

Your editorial team discussed the manuscript to determine its suitability for the Nature Portfolio Guided OA pilot. Our assessment of your manuscript takes into account several factors, including whether the work meets the **technical standard** of the Nature Portfolio and whether the findings are of **immediate significance** to the readership of at least one of the participating journals in the Nature Portfolio Guided Open Access methods cluster.

Peer review

Experts were asked to evaluate the following aspects of your manuscript:

Novelty in comparison to prior publications;

Likely audience of researchers in terms of broad fields of study and size;

Potential impact of the study on the immediate or wider research field;

Evidence for the claims and whether additional experiments or analyses could feasibly strengthen the evidence;

Methodological detail and whether the manuscript is reproducible as written;

Appropriateness of the literature review.

Editorial evaluation of reviews

Your editorial team discussed the potential suitability of your manuscript for each of the participating journals. They then discussed the revisions necessary in order for the work to be published, keeping each journal's specific editorial criteria in mind.

Journals in the Nature portfolio will support authors wishing to transfer their reviews and (where reviewers agree) the reviewers' identities to journals outside of Springer Nature. For any questions about review portability, please contact our editorial office: guidedoa@nature.com

Annotated Reviewer Reports

The editor has included some additional comments on the specific points raised by the reviewers below. However, please note that all points should be addressed in a revision, even if the editor has not specifically commented on them.

Reviewer #1

**Reviewer #1**	This reviewer has not chosen to waive anonymity. The reviewer's identity can only be shared with representatives of an established journal editorial office.
**Reviewer #1 expertise** *Summarised by the editor*	This reviewer has expertise in computational tool development, including techniques to analyze DNA methylation or deconvolute bulk datasets
**Editor's comments about this review**	While this Reviewer provided positive feedback regarding the study design and potential resource value, they highlighted the need to further discuss potential advantages or limitations of this approach and offer several suggestions to improve analyses of tissue-specific methylation patterns. Based on feedback from this reviewer, we believe it would also be necessary (for consideration at *Nature Communications*) to discuss any potential limitations regarding the generalizability of this approach based on the demographic composition of the source cohort.

Reviewer #1 comments

**Overview**	The authors present a new method for cell type deconvolution of DNA methylation data, greatly expanding the number of cell types from previous algorithms, including Houseman and other methods.<br><br>This paper represents a significant advance. The methodology is robust and the results are convincing. This paper and method promises broad application in the DNA methylation community, as virtually every human blood DNA methylation array study is likely to benefit from this software. Work and conclusions are original and solid. Previous work is appropriately credited, and appropriate context is provided.<br><br>Paper is likely to be highly cited and influence the entire EWAS field.

Specific comments

#	Reviewer comment	Editorial comment
**Major Concerns**		

1	**Line 144:** A lot of different consortium data includes both EPIC and 450k. Is there an approach here that can be applied on all the data. What about a common analysis/IDOL that includes both arrays? Is there a solution for this problem (could be valuable in some contexts)? Is this possible?	These points would only have to speculated on in the Discussion for consideration at *Communications Biology* or *Nature Communications.*
2	**Line 240:** *"Our libraries have high accuracy and minimal bias when compared to flow cytometry gold standard data."*<br>Please reference some numbers/estimates here. Progress is shown and this method represents a significant advance but no method is perfect. Please discuss pros and cons.	As mentioned in **Point #4** below, proper discussion of the context, advantages, and limitations of the method would be necessary for further consideration at *Communications Biology* or *Nature Communications*.
3	**Line 248:** Main comment: Results for specific genes are interesting but given that many of these DNA methylation sites are located in open sea regions and enhancers, a chromatin based analysis approach that does not depend on genes would be appropriate.<br><br>Why not use eFORGE/eFORGE-TF or another motif or chromatin analysis method to validate independently that these are tissue-specific elements (ideally using an enhancer mark e.g. H3K4me1)?<br><br>An example of such an analysis is appended as a pdf. The MSigDB analysis is interesting but paper would greatly benefit from an agnostic, chromatin-based method given the amount of enhancer-located sites.	Examination of tissue-specific elements using eFORGE or a related tool would be necessary for consideration at *Communications Biology* or *Nature Communications*.
4	**Line 262:** Main comment: In what way does the IDOL method compare with other methods, for example robust partial correlations? What are the advantages of this iterative method? Discuss	
**Minor Concerns**		
5	**Line 26:** Retrospective, do you mean "previous"?	Most of these minor comments reflect grammatical errors or unclear points of discussion. Please carefully proofread for clarity and grammatical errors.
6	**Line 31:** "Immune system" or "epigenetics of the immune system"?	

7	**Throughout the manuscript:** Continued use of the word “library” could potentially be confusing. As the authors will know, library is commonly used in experimental genomics with a slightly different angle and of course this is purely computational so context is different. There is a precedent in previous pipelines but potentially worth clarifying at the beginning of the text.	
8	**Line 80:** Explain how you go from 12 to 19.	
9	**Line 106:** Why is this lower number in the range (85.7) not included/mentioned in the results discussed immediately afterwards (we go from mean to median, maybe could be clarified and focus on just one measure)?	
10	**Line 117:** "slide beadchip" as a remainder of technical variation. I assume this is later removed. Please discuss/mention this.	We believe the Reviewer is asking you to clarify whether (and how) technical variability was accounted for during analysis.
11	**Line 133:** I am familiar with CpG Island shores but still not sure if north or south component has much relevance here, please explain and if not clear suggest focusing just on describing "CpG Island shores" as north and south might be confusing to non-experts.	Please either clearly define the concept of “north” and “south” shore regions, or limit the discussion to the broad concept of CpG island shores.
12	**Line 150:** Regarding the genomic context, it is not clear if the probe distribution by genomic context for IDOL libraries is any different from the array from which the data was selected… comparison to background probes on the array and some statistical test could potentially be enlightening in this respect.	
13	**line 168:** Was the flow cytometry done by this study or by a previously published study? Please clarify.	Please provide a citation (not just the accession), if relevant.
14	**Line 187:** Why was the component of nucleated red blood cells added to the myeloid granulocyte component specifically?	
15	**Line 216:** A lot of proportional findings for different diseases are reported; can one be explained in depth? Suggest to focus on at least one and describe biological interpretation in depth.	
16	**Figure 3:** As the authors know, DHSs are open chromatin, so is "open chromatin" used for ATAC-seq data? Discuss	
17	**Figure 4A**: Why R^2 0.16 for monocytes?	Please clarify why there is such a

		low correlation for monocytes in relation to the other cell types in this panel.
18	**Figure 4C:** Discuss R^2 0.65 for NK cells.	Please clarify why there is such a low correlation for NK cells in relation to the other cell types in this panel.
19	**Line 227:** change in immune proportions with age. Can part of the literature on this be referenced?	
20	**Line 239:** ">100,000 samples" Where is this number from?	
21	**Line 256:** Genes or probes?	
22	**Lines 264/265:** I'm not sure what EPX absence proves maybe this should be explained further or contextualized?	
23	**Line 268:** "than" or "from"?	
24	**Line 273:** Seems like a comma is missing.	
25	**Line 274:** can or "they can"?	
26	**Line 276:** *"Thus careful interpretation based on those estimates alone is prudent."*<br><br>Please clarify.	
27	**Line 282:** *"a careful assessment of any high basophil signal is warranted"*<br><br>Could the tool include a flag/warning for these high basophil signals as default?	
28	**Line 289:** "controlling" or "controlling for"?	
29	**Line 289:** "effects", do you mean "DNA methylation changes"?	
30	**Line 290:** "for" or "for in"?	
31	**Line 292:** "to" or "for"?	
32	**Line 302:** Long sentence *"We then compared the performance of cell estimates obtained applying our previously developed library with six cell-types available in*	

	FlowSorted. Blood.EPIC and optimized with the L-DMR IDOL library to the older 450 K array in artificial blood mixtures with predefined cell proportions."<br><br>Please clarify, I suggest breaking this statement down into shorter sentences.	
**33**	**Line 313:** *"HIV, HBV, and HBC."*<br><br>Please explain these acronyms. Do you mean HCV?	
**34**	**Line 323:** *"The reconstruction mixtures were determined by randomly generating proportions from a twelve-component Dirichlet distribution."*<br><br>Is this an *in silico* mixture or an experimental mixture?	
**35**	**Line 326:** *"Each mixture contained 1.2 µg of total DNA was estimated using the proportions included in Supplementary Table 2."*<br><br>Please clarify.	
**36**	**Line 338:** Wouldn't this sample selection based on predicted purity by your model potentially induce overfitting? Discuss.	
**37**	**Line 352:** "*and SNPs derived from Infinium Type I probes were added using the total signal intensities*."<br><br>Why did you add SNPs? Please clarify,.	
**38**	**Line 354:** "Probes corresponding to CpGs" or "measurements corresponding to probes"?<br><br>The EPIC only has ~870k CpGs	
**39**	**Line 376:** Can you show this elbow in a supplementary figure (not referenced)?	
**40**	**Reproducibility:** Work shows framework for reproducibility, given incorporation into Bioconductor pipelines presented. Sufficient reproducibility details and analyses are provided.	

Reviewer #2

**Reviewer #2**	This reviewer has not chosen to waive anonymity. The reviewer's identity can only be shared with representatives of an established journal editorial office.
**Reviewer #2 expertise** *Summarised by the editor*	This reviewer has expertise in computational tool development and deconvolution of bulk sequencing data.
**Editor's comments about this review**	This Reviewer also offers positive feedback regarding the resource value, but raises an important concern regarding whether the library was appropriately validated in an independent dataset. They also raise concerns about data availability (namely, presenting summary results) that should be addressed in a revision.
**Reviewer #2 comments**	
**Overview**	Earlier, Salas et al. created a deconvolution library (a set of CpG markers) based on the Illumina 450K array DNA methylation data that can be used to discriminate six major cell types (CD4+, CD8+, NK, B, monocytes, and neutrophils) in blood. In the current work, the authors generated a new deconvolution library based on their Illumina 850K array data on 12 immune cell types to augment the cell type discrimination capability from 6 to 12. The 12 immune cell populations were isolated and purified from the PBMCs of 56 healthy donors. This new DNA methylation dataset is a useful resource not only for deconvolution of PBMC samples but also for other applications involving DNA methylation.

Specific comments

#	Reviewer comment	Editorial comment
**Major Concerns**		
**1**	My main concern about the work is how the validation of the libraries was carried out. One of the validation datasets was the cell mixtures of the 12 populations which is fine. However, it is not clear if any of the 56 samples were set aside as an independent dataset for validation. A real dataset with known cell proportions (e.g., from FACS) is essential for this goal. The authors should use a subset of the 56 samples as the test samples or consider generating additional independent PBMC samples with both DNA methylation and cell proportion data for validation.	Inclusion of an independent dataset (including FACS data) would be necessary for consideration at *Communications Biology* or *Nature Communications*.
**2**	The authors carried out a series of validation analyses using five datasets for most of which the cell proportions are only known for the six major types, not the 12 types. Without knowing the true proportions of the 12 cell types	Please include this data (at a minimum, the summary results) along with the revised manuscript. The Reviewer also mentions a

	in each sample in those datasets, it is hard to image how one could assess the performance of the libraries on discriminating those populations. Besides the cell population mismatch problem, I do not find results for some of the validation data (e.g., the publicly available glioma, umbilical cord data, and PBMCs). Some description of those datasets and summary results should be presented and discussed.	broader point about how datasets are introduced in the Results, and quickly passed over. Please ensure that you thoroughly discuss the relevance and any insight provided through the analysis of each dataset.
**3**	Lastly, the authors also applied their libraries to 12 publicly available datasets with DNA methylation data from healthy controls and patients with diseases such as sclerosis or rheumatoid arthritis, and from pairs of monozygotic twins and dizygotic twins. Minimal descriptive results were presented for each dataset. It would be helpful to elaborate whether their analysis results recapitulated known biology or provided new insight on the underlying biology.	Please expand the Results and Discussion to comment on new or confirmatory results from each of these datasets.
**Comments Regarding Figures**		
**4**	**Figure 1:** The legend is grossly simplistic. Some elaboration on each module would be needed.	Please provide a more descriptive title. We would also recommend breaking the figure into multiple subpanels (say, Fig. 1a-c) that could be further described in the legend.
**5**	**Figure 2A (top panel):** It is not clear what the two proportional plots are referring to. The X-axis does not have a label.	
**6**	**Figure 2B:** I would not plot both training and testing results in the same plot. What were the training and testing data used in this analysis? Were the training data the 56 samples and the testing data the cell mixtures? How were the cell mixtures generated? A supplementary table listing the proportions of each cell type might be good. Why were there only a few data points in each plot?	
**7**	**Figure 3:** Some of the hypermethylated CpGs show little differential methylation patterns among the cell types. It is not clear how discriminative those CpGs are. What is the relevance of CpG genomic context, function context, and transcriptional regulatory elements of the CpGs in the context of cell type discrimination? I am not sure how informative those data are.	

8	**Figure 4:** Why did the authors only plot data for the 6 major cell types and not the 12? The predicted and true proportions of the 12 immune cell types for the cell mixtures are available.	Please include data for all 12 cell types.
9	**Figure 5:** How many of the CpGs in the libraries have also being identified by others as markers for deconvolution?	
**Minor Concerns:**		
10	It is somewhat puzzling that the number of CpGs that overlap between the “legacy” (450 IDOL-ext) and EPIC IDOL-Ext libraries is rather low (300 out of 1500). The low overlap appears to be true for other comparisons (Table 1). Any explanation would be helpful.	
11	Line 52, I would describe CIBERSORT as “support vector regression” rather than “support vector machine”.	
12	How was the number of CpGs in each library (e.g., 1200 in EPIC IDO-Ext) determined, based on an arbitrary cutoff or some kind of feature selection?	
13	I think it would be useful to include pDCs (plasmacytoid dendritic cells) in the panel should the authors consider to further expand their cell types in the future.	While we agree that expanding the panel would be useful, this point would not be necessary for *Communications Biology* or *Nature Communications.*
14	**Reproducibility:** See my comments above. I think it can be improved for reproducibility.	We believe the Reviewer’s concerns from reproducibility largely stem from the absence of summary statistics. Please also be sure to clearly state the source of each dataset used for analysis.

Open Research Evaluation	
**Data Availability**	
**Data Availability statement**	This journal strongly supports public availability of data and custom code associated with the paper in a persistent repository where they can be freely and enduringly accessed or as a supplementary data file when no appropriate repository is available. If data and code can only be shared on request, please explain why in your Data Availability statement, and also in the correspondence with your editor. For more information, please refer to https://www.nature.com/nature-research/editorial-policies/reporting-standards#availability-of-data<br><br>Please ensure that datasets deposited in public repositories are now publicly accessible, and that accession codes or DOI are provided in the "Data Availability" section. **As long as these datasets are not public, we cannot proceed with the acceptance of your paper.** For data that have been obtained from publicly available sources, please provide a URL and the specific data product name in the data availability statement. Data with a DOI should be further cited in the methods reference section.<br><br>**In particular, there is currently a reference to accession GSE167998, which is private or unavailable.**<br><br>See our policy page for details.
**Mandatory data deposition**	Mandatory data deposition is required for microarray data (which must be MIAME compliant).<br><br>For more information on mandatory data deposition policies at the Nature Portfolio, please visit http://www.nature.com/authors/policies/availability.html#data<br><br>For an up-to-date list of approved repositories for each

	mandatory data type, please visit https://www.springernature.com/gp/authors/research-data-policy/repositories/12327124<br><br>Accession code(s) for deposited data must be provided in the Data Availability statement in the final version of the paper. Failure to do so will delay publication. Please ensure data are available prior to publication.
**Flow cytometry data**	Please provide a Supplementary Figure to graphically account for all FACS sequential gating/sorting strategies, or provide gating/sorting strategies in-figure. If the former, please be sure to indicate, in the Supplementary Figure legend, which gating panel(s) correspond to which FACS data panel(s) in the manuscript figures. (For an example, please see https://www.nature.com/articles/ncomms15067#supplementary-information).
**Source data**	The following figure panels should be accompanied by the underlying source data: **Figures 2, 4, and 5, Supp. Figures 1, 4-15**.<br><br>Source data files will be mandatory prior to publication in a Nature Portfolio journal.
**Data citation**	Where datasets are hosted in public repositories that provide datasets with Digital Object Identifiers (DOIs), we encourage these datasets to be formally cited in reference lists. Citations of datasets, when they appear in the reference list, should include the minimum information recommended by DataCite and follow journal style. For example:<br><br>Hao, Z., AghaKouchak, A., Nakhjiri, N., Farahmand, A. Global Integrated Drought Monitoring and Prediction System (GIDMaPS) Data sets. *figshare*. http://dx.doi.org/10.6084/m9.figshare.853801 (2014)<br><br>Citing and referencing data in publications supports reproducible research, by increasing the transparency and provenance tracking of data generated or analysed during research. Citing data formally in reference lists also helps facilitate the tracking of data reuse and may help assign credit for individuals' contributions to research. A number of Springer Nature imprints are signatories of the Joint Declaration on Data Citation Principles, which stress the

	importance of data resources in scientific communication.
**Code Availability**	
**Code Availability**	See our policy page for details.
**Code Citation**	In addition to making the custom code available, we ask that you ensure that the version of the code/software described in the paper is deposited in a DOI-minting repository (eg, Zenodo) and that this DOI is also cited in the main Reference list. See here for details.
**Display Items**	
**Data Presentation**	Please ensure that data presented in a plot, chart or other visual representation format shows data distribution clearly (e.g. dot plots, box-and-whisker plots). When using bar charts, please overlay the corresponding data points (as dot plots) whenever possible and always for n ≤ 10. (Please see the following editorial for the rationale behind this request and an example https://www.nature.com/articles/s41551-017-0079).<br><br>For all graphs depicting a single point value (e.g., mean) with error bars, you must add individual data points or convert the graph to a boxplot or dot-plot. This applies to bar graphs and line graphs. You may wish to refer to this blog post about representing data distribution in plots (particularly for small datasets). We strongly encourage the same for plots with multiple time courses depicted. See the June 24, 2019 CommsBio editorial for more details about this policy.
**Figure Legends**	The figure legends must indicate the statistical test used. Where appropriate, please indicate in the figure legends whether the statistical tests were one-sided or two-sided and whether adjustments were made for multiple comparisons.<br><br>**Figure 5, Supplementary Figures 1a-1b, 4, 8, 9, 10a-10b, 11, 12:** Please note that the box plots need to be defined in terms of minima, maxima, centre, bounds of box and whiskers and percentile.

	**Supplementary Figures 1a-1b:** Please note that the information on whether the statistical test used was one-sided or two-sided, where appropriate, is missing. Furthermore, the panels A and B have been labelled in the legend; however, the corresponding labelling is missing from the figure panels. Please rectify this in the figure panels, accordingly.<br><br>**Supplementary Figures 3, 8, 9, 10a-10b, 12:** Please indicate the statistical test used for data analysis and where appropriate, please specify whether it was one-sided or two-sided and whether adjustments were made for multiple comparisons.<br><br>**Supplementary Figures 5 and 10:** The sub-figures A and B have been labelled in the panels; however, the corresponding labelling is missing from the respective figure legends. Please rectify this in the respective figure legends, accordingly.<br><br>**Supplementary Figure 9:** Please note that the exact p value should be provided, when possible.
**Supplementary Data**	Large datasets must be supplied as separate Supplementary Data files (in Excel or text format). Each file must be labelled as Supplementary Data 1, etc. **Please upload Supplementary Table 7 as a Supplementary Data file, separate from the Supplementary Information.**
**Research ethics**	
**Competing Interests**	In the interests of transparency and to help readers form their own judgements of potential bias, Nature Portfolio journals require authors to declare any competing financial and/or non-financial interests in relation to the work described.<br><br>Please provide a 'Competing interests' statement using one of the following standard sentences:<br>• The authors declare the following competing interests: [specify competing interests]<br>• The authors declare no competing interests.<br><br>**Competing interests have only been declared for two authors (Dr. Weincke and Kelsey); please explicitly state**

	**whether the author authors have competing interests.**<br>See our competing interests policy for further information: https://www.nature.com/nature-research/editorial-policies/competing-interests
**Research with human participants**	Research involving human research participants must have been performed in accordance with the Declaration of Helsinki . Please provide confirmation that all relevant ethical regulations were followed and that informed consent was obtained. This must be stated in the Methods section, including the name of the board and institution that approved the study protocol. See our ethics policy for details.
**Studies involving human embryos, gametes, and stem cells**	Please include an ethics statement identifying the institutional and/or licensing committees approving the experiments and describing any relevant details. The ethics statement must also confirm that informed consent was obtained from all recipients and/or donors of cells or tissues, where necessary, and describe the conditions of donation of materials for research, such as human embryos or gametes. Copies of approval and redacted consent documents may be requested by the editors.<br>We encourage authors to follow the principles laid out in the 2016 ISSCR Guidelines for Stem Cell Research and Clinical Applications of Stem Cells.
**Methods assessment and reproducibility**	
**Reproducibility**	Please state in the legends how many times each experiment was repeated independently with similar results. This is needed for all experiments, but is particularly important wherever results from representative experiments (such as micrographs) are shown. If space in the legends is limiting, this information can be included in a section titled “Statistics and Reproducibility” in the methods section.
**Statistical Evaluation**	• Wherever statistics have been derived (e.g. error bars, box plots, statistical significance) the legend needs to provide and define the n number (i.e. the sample size used to derive statistics) as a precise value (not a range), using the wording “n=X

	biologically independent samples/animals/cells/independent experiments/n= X cells examined over Y independent experiments” etc. as applicable.<br>• Please note that statistics such as error bars significance and p values cannot be derived from n<3 and must be removed in all such cases.<br>• We strongly discourage deriving statistics from technical replicates, unless there is a clear scientific justification for why providing this information is important. Conflating technical and biological variability, e.g., by pooling technically replicates samples across independent experiments is strongly discouraged. (For examples of expected description of statistics in figure legends, please see the following https://www.nature.com/articles/s41467-019-11636-5 or https://www.nature.com/articles/s41467-019-11510-4).<br>• All error bars need to be defined in the legends (e.g. SD, SEM) together with a measure of centre (e.g. mean, median). For example, the legends should state something along the lines of “Data are presented as mean values +/- SEM” as appropriate.<br>• All box plots need to be defined in the legends in terms of minima, maxima, centre, bounds of box and whiskers and percentile.<br>• For null hypothesis testing, please indicate the test statistic (e.g. F, t, r) with confidence intervals, effect sizes, degrees of freedom and P values noted.<br>• Please provide the test results (e.g. P values) as exact values whenever possible and with confidence intervals noted.
**Digital image integrity**	Please pay close attention to our Digital Image Integrity Guidelines. Also ensure that you retain unprocessed data and metadata files after publication, ideally archiving data in perpetuity, as these may be requested during the peer review and production process or after publication if any issues arise.
**Methods Descriptions**	The Methods must contain sufficient detail such that the work could be repeated. It is preferable that all key methods be included in the main manuscript, rather than in the Supplementary Information.

	Nature Portfolio journals encourage authors to share their step-by-step experimental protocols on a protocol sharing platform of their choice. Where such protocols are available, please provide a DOI or other citation details in the paper. The Nature Portfolio's *Protocol Exchange* is a free-to-use and open resource for protocols; protocols deposited in *Protocol Exchange* are citable and can be linked from the published article. More details can found at www.nature.com/protocolexchange/about.
**Study design and reporting**	We have included as an attachment to the decision letter a version of your Reporting Summary with a few notes. This is mainly for your information, but we hope it is helpful when preparing your revised manuscript. If you decide to resubmit the manuscript for further consideration, please be sure to include an updated Reporting Summary.
